# Real-time observation of non-equilibrium phonon-electron energy and angular momentum flow in laser-heated nickel

**DOI:** 10.1126/sciadv.adj2407

**Published:** 2024-01-31

**Authors:** Vishal Shokeen, Michael Heber, Dmytro Kutnyakhov, Xiaocui Wang, Alexander Yaroslavtsev, Pablo Maldonado, Marco Berritta, Nils Wind, Lukas Wenthaus, Federico Pressacco, Chul-Hee Min, Matz Nissen, Sanjoy K. Mahatha, Siarhei Dziarzhytski, Peter M. Oppeneer, Kai Rossnagel, Hans-Joachim Elmers, Gerd Schönhense, Hermann A. Dürr

**Affiliations:** ^1^ Department of Physics and Astronomy, Uppsala University, 751 20 Uppsala, Sweden.; ^2^ Deutsches Elektronen-Synchrotron DESY, 22607 Hamburg, Germany.; ^3^ Institut für Experimentalphysik, Universität Hamburg, 22761 Hamburg, Germany.; ^4^ Ruprecht Haensel Laboratory, Deutsches Elektronen-Synchrotron DESY, 22607 Hamburg, Germany.; ^5^ Institut für Experimentelle und Angewandte Physik, Christian-Albrechts-Universität zu Kiel, 24098 Kiel, Germany.; ^6^ Institut für Physik, Johannes Gutenberg-Universität Mainz, 55128 Mainz, Germany.

## Abstract

Identifying the microscopic nature of non-equilibrium energy transfer mechanisms among electronic, spin, and lattice degrees of freedom is central to understanding ultrafast phenomena such as manipulating magnetism on the femtosecond timescale. Here, we use time- and angle-resolved photoemission spectroscopy to go beyond the often-used ensemble-averaged view of non-equilibrium dynamics in terms of quasiparticle temperature evolutions. We show for ferromagnetic Ni that the non-equilibrium electron and spin dynamics display pronounced variations with electron momentum, whereas the magnetic exchange interaction remains isotropic. This highlights the influence of lattice-mediated scattering processes and opens a pathway toward unraveling the still elusive microscopic mechanism of spin-lattice angular momentum transfer.

## Teaser

Non-equilibrium phonon-electron scattering leads to momentum-dependent transient electron and spin populations in Ni.

## INTRODUCTION

The ability to drive a system far out of equilibrium by absorbing a femtosecond (fs) laser pulse provides access to dynamical phenomena (*1*–*6*). Such far-from-equilibrium dynamics offers non-thermodynamic pathways for the ultrafast control of materials’ properties before thermalization on longer timescales is reached (*7*, *8*). While the initial ultrafast laser heating of the electronic system and the subsequent energy transfer to other degrees of freedom (spin and lattice) can be studied in so-called pump-probe measurements (*9*–*14*), the understanding of such strongly non-equilibrium dynamics in solids is still very limited. Relying on the quasiparticle description of electronic, spin, and lattice excitations, temperatures are often assigned to the individual quasiparticle reservoirs (*15*). It is assumed that the quasiparticle subsystems are each in separate equilibrium at all times and reach global equilibrium by exchanging heat (*15*, *16*).

The ensemble-averaged non-equilibrium heat exchange between electron, spin, and lattice quasiparticles can be described by a rate equation treatment within the three-temperature model which leads to different demagnetization timescales depending on pump fluence (*10*). However, it has so far been difficult to identify the quasiparticle states that are directly involved in energy, momentum, and, for magnetic systems, angular momentum transfer processes. Several attempts have been made to identify points in energy and momentum space that facilitate electronic spin-flip processes deemed necessary for ultrafast demagnetization of ferromagnets (*17*, *18*). It remains ambiguous which phonons take up spin angular momentum in a non-equilibrium demagnetization process (*19*, *20*). While the excited electron system reaches rapidly a uniform electron temperature through fast electron-electron scattering within a few hundred femtoseconds (*11*, *21*), phonon equilibration can take tens of picoseconds (*22*–*25*). In addition, the energy transferred from electrons to certain phonon modes can be donated back to the electrons much faster (*24*, *25*) thus increasing the potential complexity of non-equilibrium pathways toward final equilibrium.

Here, we aim to identify the quasiparticle states responsible for energy and angular momentum redistribution between the electronic system and the crystal lattice. We expect that this provides the experimental benchmarks for modeling and ultimately understanding the enigmatic ultrafast spin-lattice angular momentum transfer process. Traditionally, this has been attempted by following the energy transfer from the electronic system to the lattice. However, in this case, many different scattering processes between electrons, spins, and lattice excitations prevent unambiguous identification so far, and mainly ensemble-averaged information via the three-temperature model is available. In the present paper, we approach this topic from a different angle. On the basis of our previous observation that non-equilibrium phonon modes are populated first, we observe in real time how these phonon modes scatter with valence electrons and affect their magnetic moments. We show that these non-equilibrium scattering processes can be unambiguously separated for the ensemble-averaged dynamics.

**Fig. 1.**
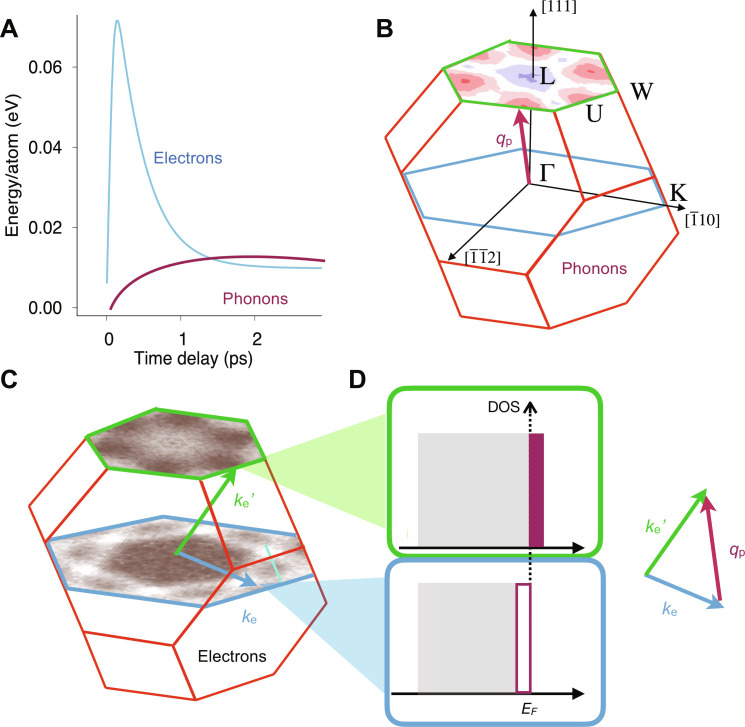
Illustration of phonon-electron energy transfer in laser-excited nickel. (**A**) Energy in the laser-excited electronic system (blue line) is transferred to phonon modes at the Brillouin zone (BZ) boundary (purple line) via electron-phonon (e-p) coupling (*24*). When the electronic energy becomes less than the energy in a particular phonon mode, p-e energy back-transfer can occur. (**B**) Calculated non-equilibrium energy distribution of BZ-boundary phonons (red: more; blue: less; see Materials and Methods and fig. S13) compared to equilibrium conditions (*24*). The purple arrow indicates a phonon momentum vector, **q**_p_, in the BZ of nickel. (**C**) Measured photoemission intensity maps near the Fermi level, *E*_F_, in the BZ of Ni within the L-W-U (green border) and Γ-K planes (blue border), respectively. White indicates higher and brown lower intensity. (**D**) Illustration of the change in electron occupation of states upon absorption of phonons with momentum, **q**_p_, and scattering of electrons from states with momentum vector, **k**_e_, to unoccupied states at **k**_e_′. The vector diagram illustrates momentum conservation.

Our approach is depicted in Fig. 1. During the first picosecond after fs laser heating, phonon modes at the Brillouin zone (BZ) boundary become populated first, while the lower-energy BZ center phonon modes become populated only at much later times (*24*). When the energy stored in the non-equilibrium population of BZ-boundary phonon modes exceeds that of the electronic system, a back-transfer becomes energetically favorable as displayed in Fig. 1A (*24*, *25*). We use time- and angle-resolved photoemission spectroscopy (tr-ARPES) to directly identify the underlying energy back-transfer processes. These are based on absorbing a non-equilibrium zone-boundary phonon (Fig. 1B) with momentum, **q**_p_, and energy, *E*_p_, (see Materials and Methods) while scattering electrons from occupied (momentum **k**_e_ and energy *E*_e_) to unoccupied states (**k**_e_′ and *E*_e_′) by conserving momentum (**k**_e_ + **q**_p_, = **k**_e_′) and energy (*E*_e_ + *E*_p_ = *E*_e_′) as shown in Fig. 1 (C and D).

Relevant BZ-boundary phonons in Ni have energies of typically 25 to 30 meV (*24*) and, therefore, we expect to see differences in band occupations on such energy scales and for electronic momentum values that are separated by a BZ-boundary phonon momentum. We report tr-ARPES measurements that detect such changes in the band-filling of laser-heated Ni using radiation from the FLASH free-electron laser to cover the complete Ni BZ. Different electron band occupations are quantified by a transient, electronic state-dependent chemical potential. We identify several pairs of Bloch states, separated by the momentum of a zone-boundary phonon, whose transient chemical potentials differ approximately by the phonon energy. This provides the direct visualization of p-e scattering processes in real time. In addition, we find an unexpected fluence dependence of this process that points to a competition between electron-phonon energy transfer. We show that this has direct consequences for the state-resolved magnetic moment dynamics deviating from the generally assumed global dynamics averaged over the whole ensemble.

## RESULTS

We measured tr-ARPES from 2- and 9-nm-thick Ni(111) films grown on W(110) at the FLASH free-electron laser in Hamburg (see Materials and Methods). Our main goal is to identify the p-e scattering processes leading to energy and angular momentum redistribution between the phonon and the electronic system. To achieve this, we need to identify *k*-points in the Ni BZ that satisfy the characteristics described in Fig. 1 for non-equilibrium phonon-electron energy transfer processes. The Ni electronic structure is characterized by itinerant 4*sp* states whose energy disperses strongly with electron momentum and 3*d* bands with flat energy dispersions. The latter is split by the exchange interaction into fully occupied Bloch states of majority-spin character and partially occupied minority-spin states. The difference in occupation numbers represents the size of the Ni magnetic moment. Ni 4*sp* bands display a spin splitting only via hybridization with the 3*d* states. Ni is thought to demagnetize both in equilibrium and following ultrafast laser excitation by a reduction of the 3*d* exchange splitting and a corresponding redistribution of the electron occupation between majority and minority states (*11*, *21*, *26*–*29*).

We study electronic bands in the L-W-U and Γ-K planes shown in Fig. 1 that are separated by zone-boundary phonon wave vectors. We include representative ARPES datasets and band structure calculations in fig. S1. The Ni electronic structure of the L-W-U plane is largely characterized by exchange split 3*d* bands straddling the Fermi level, *E*_F_. Only near the L point are itinerant *sp* bands hybridizing with the magnetic 3*d* states. The situation is different in the Γ-K plane where a large proportion of the band structure is characterized by the *sp* band crossing the Fermi level. Only along the $\Gamma -d_{11\bar{2}}$ direction, we find strongly dispersing 3*d* bands and the minority-spin 3*d* band crossing the Fermi level in agreement with previous studies (*11*, *21*, *26*–*29*). We note that $d_{11\bar{2}}$ is not a high-symmetry point. Instead, it describes a minority-spin hole pocket located approximately halfway between X and L points (*29*). Note that the crossing points between *sp* and 3*d* states can contain so-called spin-orbit hotspots, i.e., points where spin-orbit coupling mixes majority- and minority-spin characters. It is thought that these hotspots play a crucial role in non-equilibrium angular momentum exchange between the electronic system and the lattice (*17*, *18*).

**Fig. 2.**
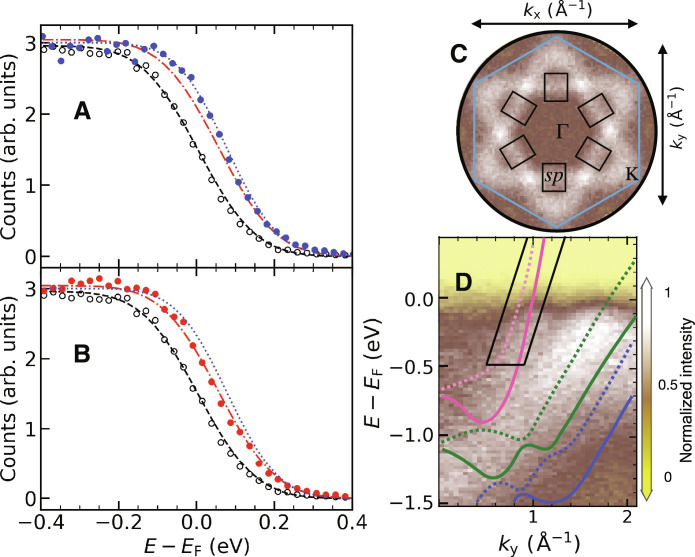
Population dynamics of *sp* bands. Tr-ARPES data for 2-nm Ni(111)/W(110) obtained for pump fluences of (**A**) 2.2 mJ/cm^2^ (blue solid circles and lines) and (**B**) 3.7 mJ/cm^2^ (red solid circles and lines) measured at delay times of 1.8 ps. Open black circles and lines correspond to spectra obtained before the arrival of the pump pulses (−0.6-ps time delay). Lines are fits to the data using a Fermi-Dirac distribution function including a chemical potential shift and a constant *sp* density of states across the shown *E*-*E*_F_ energy range. (**C**) ARPES data of the integrated intensity near the Fermi level, *E*_F_, in the (*k*_x_, *k*_y_) plane (see Fig. 1). The black rectangles mark the integration region within which the spectra shown in (A) and (B) were obtained. (**D**) The ARPES intensity as *E*-*E*_F_ versus *k*_y_ along the Γ-K direction (see Fig. 1). The black parallelogram shows the *E*-*E*_F_ dependence of the (*k*_x_, *k*_y_) integration areas (black rectangles) in (C).

Figure 2 (extended datasets are shown in figs. S2 to S4) shows the case of the *sp* band Fermi crossing along the Γ-K direction. The tr-ARPES data were evaluated for the (*k*_x_, *k*_y_) regions shown in Fig. 2C. Since the *sp* bands have a steep dispersion, we shifted the *k*-integration regions for different *E*-*E*_F_ values according to the observed dispersion of Δ*E*/Δ*k* = 1.65 eVÅ (see Fig. 2D). We note that the *k*-integration region has been chosen wide enough to average over the exchange split majority- and minority-spin *sp* bands. The data in Fig. 2 can then be fitted by a Fermi-Dirac distribution function with the electronic temperature, *T*_e_, and the chemical potential, μ, as parameters (see Supplementary Materials). We assume a constant *sp* band density of states for the *E-E*_F_ values in Fig. 2. The resulting delay-time dependencies of the fit parameters will be discussed below. Here, it is important to highlight the clearly visible chemical potential shift. This effect can be unambiguously separated from the observed broadening due to increased electronic temperatures, *T*_e_, after optical excitation (*11*, *21*, *28*). We also point out that the chemical potential shift displays an interesting fluence dependence that is contrary to expectations, i.e., the chemical potential shift is larger at 2.2 mJ/cm^2^ (Fig. 2A) than at 3.7 mJ/cm^2^ (Fig. 2B). We will discuss this behavior below as a competition between p-e energy transfer and electron-electron scattering.

**Fig. 3.**
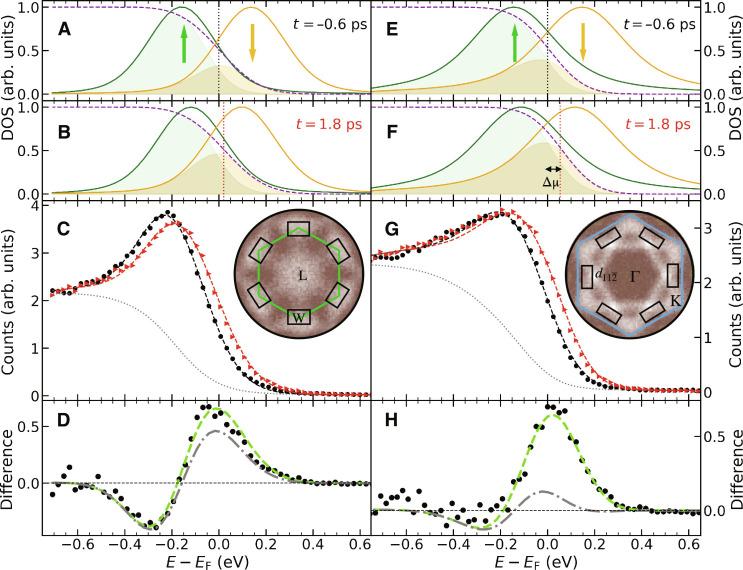
Dynamics of 3*d* bands at selected *k*-points. Tr-ARPES data for 2-nm Ni(111)/W(110) and 3.7 mJ/cm*^2^* pump fluence (**A** to **D**) at the W point and (**E** to **H**) at the $d_{11\bar{2}}$ point. Spectra obtained at time delays of 1.8 and −0.6 ps, i.e., before time zero, are shown as red and black solid symbols, respectively. Fits of the spectral lineshapes after background [dotted lines in (C) and (G)] subtraction (*30*) are shown in (A) and (E) and (B) and (F) for −0.6 and 1.8 ps, respectively. The Lorentzian functions represent the exchange split 3*d* bands, the purple dashed lines are the Fermi-Dirac functions and the vertical dotted lines mark the position of the chemical potentials. The resulting fits to the measured spectra are included in (C) and (G) as dashed lines. The shaded areas indicate the occupied parts of the respective spin states. (D) and (H) show the difference spectra (solid symbols) of the data in (C) and (G). The (green) dashed lines are the fits to the data, while the gray dash-dotted lines represent the calculated spectra for zero chemical potential change. The obtained fit parameters are summarized in Fig. 4. The insets in (C) and (G) display the measured tr-ARPES intensities around the Fermi level in the respective (*k*_x_, *k*_y_) planes of the BZ and the regions over which the spectra shown in (C) and (G) were averaged.

The situation is more complex for the 3*d* bands shown in Fig. 3, where also the change in exchange splitting and spin-dependent band occupation needs to be taken into account. Figure 3 (A to D) shows the dynamics at the W point (see Fig. 1B and inset of Fig. 3C for the position in momentum space). We model the exchange split 3*d* bands as illustrated in Fig. 3A before and in Fig. 3B 1.8 ps after a 3.7 mJ/cm^2^ pump pulse. Ultrafast demagnetization (*9*) is approximated by asymmetrical energy shifts where majority-spin states below *E*_F_ move twice as fast toward *E*_F_ as minority-spin states located above *E*_F_ (*27*). This leads to an excellent match (dashed lines) with the measured tr-ARPES data (symbols) (Fig. 3C) considering a time-independent background (*21*, *30*) (dotted line). Figure 3D illustrates the reason for the observed spectral changes. The intensity reduction observed near *E-E*_F_ = −0.3 eV reflects the shift of the occupied majority-spin state toward *E*_F_. Consequently, it becomes partially depopulated as shown by the (green) shaded areas beneath the majority peak (solid green line) in Fig. 3 (A and B). The increase of intensity near *E*_F_ is mainly due to the population transfer (yellow shaded area) from the majority to the minority-spin states shifted to lower energy (yellow solid line). At the W point shown in Fig. 3 (A to D) these processes are largely unaffected by a change in chemical potential as indicated by the gray dash-dotted line (Δμ = 0 meV) being nearly identical to the green dashed line (Δμ = 20 meV) in Fig. 3D. We used electronic temperatures that were obtained from data shown in Fig. 2 measured at identical pump fluences.

**Fig. 4.**
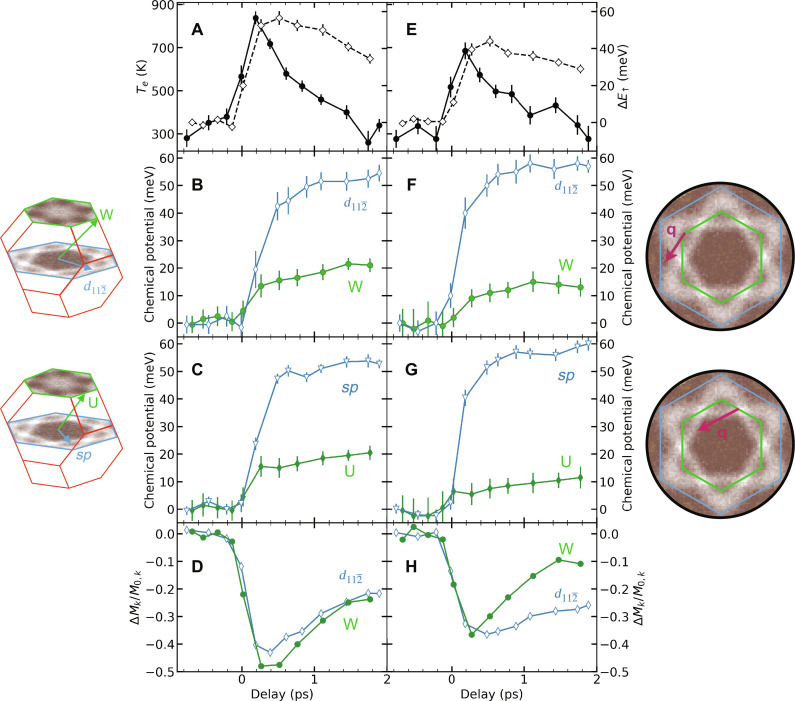
Temporal evolution of temperature, magnetization, and chemical potential at selected *k*-points. (**A**) Laser-induced transient electronic temperature (solid symbols and lines) and average energy shift of the majority 3*d* bands (see fig. S4) due to ultrafast demagnetization (open symbols and dashed lines) for a pump fluence of 3.7 mJ/cm^2^. (**B** and **C**) Chemical potential changes versus pump-probe time delay at the momentum vectors indicated in the left insets. (**D**) Magnetic moment change, *M*_k_, at the respective *k*-points. *M*_0,k_ are the moments before laser excitation. The projection of the electron momentum vectors onto the Γ-K and L-W-U planes are shown in the right insets. The indicated purple wave vectors, **q**, represent BZ-boundary phonon modes. (**E** to **H**) Same as (A) to (D) but for a pump fluence of 2.2 mJ/cm^2^.

In contrast, the tr-ARPES results at the $d_{11\bar{2}}$ point (see Fig. 1B and inset of Fig. 3G for the position in momentum space) shown in Fig. 3 (E to H) display characteristic differences. While the changes in exchange splitting are identical to those at other *k*-points (see fig. S6), the chemical potential shifts by up to Δμ = 60 meV as illustrated by the Fermi-Dirac distribution curves (purple dashed lines) in Fig. 3 (E and F). This results in a negligible intensity reduction near *E-E*_F_ = −0.3 eV and a stronger intensity increase at *E*_F_ in Fig. 3H. The latter is strongly affected by the energy position of the chemical potential (see green dashed and gray dash-dotted lines in Fig. 3H). We find similar characteristics as for the W and $d_{11\bar{2}}$ points in Fig. 3 for all analyzed *k*-points (see figs. S2 and S3 for 3.7 and 2.2 mJ/cm^2^ pump fluences, respectively). The resulting *k*-dependent values for the delay time dependence of the local chemical potential, electronic temperature, exchange splitting, and spin-dependent band occupation are summarized in Fig. 4 and represent the central result of this paper.

## DISCUSSION

The results shown in Fig. 4 (A and E) reflect the commonly accepted ultrafast dynamics that occur in ferromagnetic Ni (*9*, *11*, *21*, *28*). Laser excitation deposits energy into the electronic system, which upon thermalization leads to an increase in the electronic temperature, *T*_e_. We find maximum *T*_e_ values of 840 ± 20 K and 700 ± 30 K for pump fluences of 3.7 mJ/cm^2^ (Fig. 4A) and 2.2 mJ/cm^2^ (Fig. 4E), respectively. This leads to a demagnetization via the transfer of energy and angular momentum to the lattice (*19*, *20*) and the excitation of magnons (*21*, *31*). The observed changes in Fig. 3 (see also fig. S6) of the majority-spin band binding energy reflects the ultrafast collapse of the magnetic exchange splitting (*11*, *21*, *28*, *32*) and an associated repopulation of majority/minority-spin bands (see Fig. 3) that is believed to be responsible for ultrafast demagnetization in Ni (*10*).

Here, we report the observation of a momentum-dependent change in band-filling that we describe as the buildup of quasi-chemical potentials. Spin-integrated results are summarized in Fig. 4 (B, C, F, and G) for pump fluences of 3.7 and 2.2 mJ/cm^2^, respectively. The buildup of such chemical potential changes is slightly delayed compared to the increase in electronic temperature and to the energy shift of the majority-spin 3*d* bands. The changes in the momentum-dependent chemical potential also persist up to the longest time delays measured, outlasting in particular the picosecond timescale remagnetization and electronic temperature decay, thus, indicating a different physical origin.

Figure 4 (D and H) displays the dynamics of momentum-dependent magnetic moments, Δ*M*_k_, at the respective *k*-points. Extended datasets are shown in fig. S14. Δ*M*_k_ was evaluated from the band occupations *N*_maj,min_ of majority- and minority-spin bands in Fig. 3 as $\frac{\Delta M_{k}}{M_{0,k}}=\frac{N_{\text{maj}}-N_{\text{min}}}{N_{0,\text{maj}}-N_{0,\text{min}}}$ , where *M*_0_ and *N*_0_ represent the magnetic moments and occupation numbers, respectively, before laser excitation. The results displayed in Fig. 4 (D and H) and fig. S14 clearly show that the magnetization dynamics at the investigated *k*-points is nonuniform, i.e., different *k*-points contribute differently to the total magnetic moment changes. This is in contrast with the observed changes in magnetic exchange splitting (fig. S6) that are the same for all measured *k*-points. We will discuss below that the observed changes are compatible with a spin-dependent energy transfer between lattice and electronic system.

It has been argued that ultrafast demagnetization of Ni is accompanied by a valence band narrowing observed in transient 2*p*-3*d* x-ray absorption (*33*, *34*) and ascribed to pump-induced non-equilibrium electron populations (*35*). This has more recently been confirmed experimentally and theoretically described as ultrafast changes in electronic correlations (*36*). Such changes in the Ni valence bands could possibly influence the observed chemical potential shifts. We have, therefore, probed the influence of both effects on Ni 3*p* core levels for 3.7 mJ/cm^2^ (see fig. S7). We observed a shift of the 3*p* core levels toward the Fermi level by 17 ± 3 meV, which is very close to the chemical potential shift of d bands at the U and W points. However, the observed core-level shift is smaller than the chemical potential changes observed for *sp* bands and at the $d_{11\bar{2}}$ point. Since core levels probe changes of the electronic system averaged over of the whole valence band (*37*), valence band narrowing related to the collapsed 3*d* exchange splitting and electronic correlations cannot explain the observed chemical potential changes in Fig. 4.

The enhanced chemical potentials of the *sp* bands and at the $d_{11\bar{2}}$ point indicate that more electrons reside in these states for delay times longer than ~0.5 ps (see Fig. 4). It is a possible option that these extra electrons are photoexcited in the W(110) substrate and reach the surface of our 2-nm-thick Ni(111) films via superdiffusive electronic currents (*38*) since also part of the W substrate is heated by the pump laser pulses. We performed additional experiments for 9-nm-thick films (see fig. S8) and found nearly identical chemical potential changes compared to the 2-nm films for the same absorbed pump fluences, i.e., for conditions leading to similar electronic temperature rises. Since the grazing incidence pump laser penetration depth is around 13 nm (*21*), we expect less heating of the W substrate for the thicker 9-nm Ni film. Consequently, fewer electrons would accumulate and be visible in surface-sensitive tr-ARPES than for the thinner 2-nm film under otherwise identical conditions. This observation clearly rules out the effect of superdiffusion for any surface accumulation of electrons possibly resulting in an electronic state-dependent band-filling. In addition, we would expect purely electronic transport phenomena to evolve in a similar way to the electronic temperature, which is clearly not the case for the chemical potential evolution shown in Fig. 4.

The observed step-like chemical potential changes, i.e., a sudden rise up to ~0.5 ps and a nearly constant value thereafter, require a non-electronic driving force to maintain the observed population imbalance within the Ni BZ. Hot non-equilibrium phonons could be such a force counteracting the equilibrating effect of an elevated electronic temperature, i.e., electron-electron scattering processes. Studies of non-equilibrium phonon dynamics show that BZ-boundary phonon modes are preferentially populated and essentially retain their energy for all times considered in the present paper (*24*). This is the scenario depicted in Fig. 1. In the following, we argue that we have identified two scattering channels through which energy can be transferred back to the electronic system.

Electronic states at the W and $d_{11\bar{2}}$ points as well as at the U point and for *sp* bands are separated in momentum space by zone-boundary phonon momenta, **q**_p_ (purple arrow in Fig. 1B). Absorption of a phonon with momentum **q**_p_ can scatter electrons from states at W to $d_{11\bar{2}}$ points and from U to *sp* bands. Since our system is far from equilibrium after the pump pulse, a large number of such scattering processes can take place and consequently electronic states at $d_{11\bar{2}}$ and *sp* bands are populated up to an energy of the phonon energy *E*_p_ of about 25 to 30 meV (*24*) averaged over the ensemble of scattered electrons. This overpopulation agrees well with the observed chemical potential differences shown in Fig. 4. We note that the changes in band-filling are smaller in Ni than in other materials (*39*, *40*) due to the smaller phonon energies.

The observed chemical potential changes shown in Fig. 4 scale inversely with the pump fluences, i.e.*,* they are larger at 2.2 mJ/cm^2^ as compared to 3.7 mJ/cm^2^. This points to a competing mechanism that counteracts the chemical potential buildup via phonon scattering. This may be due to the higher electronic temperatures at larger pump fluences, leading to more electronic scattering processes that can equilibrate population differences throughout the BZ. We can provide additional evidence for such a competition by investigating the involved timescales. While we do not obtain phonon-electron scattering times, τ_e-p_, directly in our experiments, it is possible to estimate them from high-resolution ARPES experiments (*29*). In Ni, so-called kinks due to electron-phonon interactions have been observed near the Fermi level for minority-spin 3*d* bands, and from the measured lifetime broadening (*29*), we can estimate τ_e-p_ ~ 100 fs. The competing process would be electron-electron scattering that leads to an increased lifetime broadening as observed in our tr-ARPES measurements. From the fitted 3*d* bandwidths at various *k*-points (see fig. S11), we obtain lifetime broadening increases of 20 to 40 meV and 40 to 50 meV for fluences of 2.2 and 3.7 mJ/cm^2^, respectively. These values correspond to electronic scattering times, τ_e-e_, of approximately 15 fs, i.e., they are much faster than the typical phonon-electron scattering times. It is, therefore, possible that phonon-electron energy transfer processes can be interrupted by faster electronic scattering events, especially at higher fluences when more electronic scattering takes place. Furthermore, the BZ-boundary modes are the first phonons that become populated (*24*) and are very likely the reservoirs of angular momentum transferred from the spin system during ultrafast demagnetization (*20*).

The changes in momentum-dependent magnetic moments observed in Fig. 4 (D and H) demonstrate that the p-e energy transfer is also spin-dependent. Demagnetization signified by a reduction of Δ*M*_k_ corresponds to electronic spin flips that increase the minority band occupation at the cost of that of majority-spin states. For a pump fluence of 3.7 mJ/cm^2^ Δ*M*_k_, the $d_{11\bar{2}}$ point largely follows those at W and U. However, the situation is very different at a lower fluence of 2.2 mJ/cm^2^ where the $d_{11\bar{2}}$ point dynamics remains mainly unaltered compared to the higher fluence but the dynamics at W and U are considerably smaller. The strong demagnetization at $d_{11\bar{2}}$ requires a transfer of minority spins from other *k*-points such as W and U. This is only possible via the uninterrupted p-e scattering described above.

Our results show the transient buildup of electronic populations in bands at different points in the BZ following ultrafast demagnetization of ferromagnetic Ni. These effects have been overlooked in previous tr-ARPES studies (*11*, *21*, *28*) but are unambiguously separated from possible pump-induced space charge effects by probing many different momentum points simultaneously using momentum microscopy at suitably high photon energy that allows covering a substantial part of the Ni BZ. Our results establish the previously proposed (*24*) energy back-transfer from transiently excited zone-boundary phonons to the electron system to be spin-dependent. Pump fluence-dependent measurements indicate that this energy back-transfer competes with another channel, possibly caused by quasiparticle scattering within the electronic system. The latter is ultimately responsible for achieving thermal equilibrium after transient phonon populations have decayed. We expect these energy relaxation channels to be operative in most materials under non-equilibrium conditions. 

## MATERIALS AND METHODS

### Ni sample growth and characterization

Ni(111) films of 2 and 9 nm thickness were grown on a W(110) single crystal by molecular beam epitaxy using e-beam evaporators at a base pressure of 5 × 10^−10^ mbar. The W(110) substrate was cleaned via several cycles of annealing (1200°C) in the presence of oxygen (1 × 10^−7^ mbar) followed by flashing to 2200°C. The deposition of nickel was initiated right after the high-temperature flash, so the substrate was cooling down during the growth of the first Ni layers. The deposited Ni(111)/W(110) films were post-annealed at 400°C for a duration of ~5 min and the quality of the deposited films was checked with low energy electron diffraction (LEED). Fresh films were deposited every ~24 hours or earlier in case surface contamination could be detected.

All data shown in the main paper were taken for 2-nm Ni(111)/W(110) films. We also measured 9-nm Ni(111)/W(110) films (see Supplementary Materials and fig. S8) to rule out the contributions from superdiffusive spin transfer to the results shown here.

### Time-resolved angle-resolved photoemission spectroscopy

The tr-ARPES measurements were performed at the PG2 beamline of the FLASH free-electron laser at DESY/Hamburg using the HEXTOF end-station (*41*). A momentum microscope with photoelectron time-of-flight analysis measures three-dimensional photoelectron intensity as a function of binding energy, *E-E*_F_, relative to the Fermi level, for electron momenta (*k*_x_, *k*_y_) roughly parallel to the sample plane. The photoelectron momentum perpendicular to the sample surface was varied via the selected FLASH photon energy to probe ARPES from the L-W-U (74 eV) and Γ-K (134 eV) BZ planes shown in Fig. 1C. The instrument provides a momentum resolution of ~0.05 Å^−1^ and covers a *k*_x,y_ range of 4 Å^−1^ diameter. The momentum component perpendicular to the surface, *k*_z_, was calculated considering an inner potential of 13.3 eV for free-electron final states (*29*). The combined photon and photoelectron energy resolution was determined to be 200 ± 4 meV as the full width at half maximum of a Gaussian function convoluting a Fermi-Dirac distribution fitted to the unpumped ARPES data for Ni *sp* bands crossing the Fermi level at the *k*-points indicated in Fig. 2. We used optical laser pulses of 1.2-eV energy and ~100-fs duration to excite the Ni electronic system. The FLASH pulse length was ~160 fs. Pump and probe pulses were incident on the sample collinearly at an angle of 22° relative to the sample surface. The pump and probe focal spot sizes on the sample were determined to be 100 × 200 μm^2^ and 50 × 100 μm^2^, respectively. P-polarization was used for both pump and probe beams. This enabled us to measure the pump-probe sum frequency–assisted photoemission signal (*42*) for monitoring the temporal pump-probe overlap and determine the combined temporal resolution to 227 ± 2 fs (see Supplementary Materials and figs. S9 and S10). The pump fluences used for the shown experiments were determined to be 2.2 and 3.7 mJ/cm^2^. All measurements shown were performed at room temperature. The ARPES spectra shown here are averaged over equivalent points in the (*k*_x_, *k*_y_) region of the BZ to improve the signal-to-noise ratio. It is important to point out that, for the selected *k*-points in Fig. 3 and figs. S2B and S3B, it is possible to obtain the population of majority and minority-spin 3*d* states without the need for cumbersome photoelectron spin analysis. This is enabled by their particular energetic position below and above the Fermi level, respectively. At all other *k*-point photoelectron spin analysis would be required which is beyond the scope of this paper.

### Theory

Our recently developed non-equilibrium theory of the wave vector–dependent ultrafast electron and lattice dynamics (*23*) has been extended here to explicitly provide the energy transfer between the laser-excited electrons and the lattice. The main features are the phonon branch and wave vector dependence of electron-phonon coupling and explicit inclusion of anharmonic effects describing phonon-phonon scattering events. We model the non-equilibrium variation of the wave vector–dependent phonon populations (*24*). Our kinetic theory captures the full transient dynamics of the non-equilibrium phononic populations. The rate of exchange that defines the time evolution of the non-equilibrium energy flow between the electronic system and the different phonon modes after laser excitation is calculated by numerically solving the following rate equations

$$\frac{\partial E_{e}}{\partial t}=\sum_{q,v}\hbar \omega_{\nu }\left(q\right)\gamma_{\nu }\left(q,E_{e},t\right)\left[n_{\nu }\left(q,E_{l}^{q}\right)-n_{\nu }\left(q,E_{e}\right)\right]+P\left(t\right)$$ (1)

$$\frac{\partial E_{\nu }^{q}}{\partial t}=-\hbar \omega_{\nu }\left(q\right)\gamma_{\nu }\left(q,E_{e},t\right)\left[n_{\nu }\left(q,E_{l}^{q}\right)-n_{\nu }\left(q,E_{e}\right)\right]+\frac{\partial E^{p-p}}{\partial t}\;\text{for}\;q=q_{1},\cdots ,q_{N}$$ (2)

where $n_{\nu }\left(q,E_{l}^{q}\right)$ is the out-of-equilibrium phonon population of phonon mode ***q*** with branch ν, with $E_{l}^{q}$ being the time-dependent amount of energy stored in this particular mode (*24*). *P*(*t*) is the pump laser field that generates the non-equilibrium electronic distribution. γ_ν_(***q***, *E*_e_, *t*) is the phonon linewidth due to e-ph scattering, which depends explicitly on the phonon mode, electronic spin degrees of freedom, and electronic energy, *E*_e_. It is therefore a time-dependent quantity. Note that, while the first terms on the right-hand side of Eq. 2 define the energy flow due to e-p interaction, the term $\frac{\partial E^{p-p}}{\partial t}$ defines the energy flow due to p-p scattering processes and explicitly accounts for the system anharmonicities (*23*). To obtain a full solution of the non-equilibrium model defined by Eq. 2, we compute all required material-specific quantities using spin-polarized density functional theory and solve Eqs. 1 and 2 numerically (*23*). The resulting transient phonon populations 1 ps after laser excitation relative to the thermal equilibrium are displayed in fig. S13 for the L-W-U and Γ-K planes (see Fig. 1B).

## Supplementary Materials

### This PDF file includes:

Supplementary Text Figs. S1 to S14 References

## References

[1] A. Kimel, A. Kirilyuk, P. A. Usachev, R. V. Pisarev, A. M. Balbashov, T. Rasing, Ultrafast non-thermal control of magnetization by instantaneous photomagnetic pulses. Nature 435, 655–657 (2005). 15917826 10.1038/nature03564

[2] F. Schmitt, P. S. Kirchmann, U. Bovensiepen, R. G. Moore, L. Rettig, M. Krenz, J.-H. Chu, N. Ru, L. Perfetti, D. H. Lu, M. Wolf, I. R. Fisher, Z.-X. Shen, Transient electronic structure and melting of a charge density wave in TbTe_3_. Science 321, 1649–1652 (2008). 18703710 10.1126/science.1160778

[3] V. R. Morrison, R. P. Chatelain, K. L. Tiwari, A. Hendaoui, A. Bruhacs, M. Chaker, B. J. Siwick, A photoinduced metal-like phase of monoclinic VO2 revealed by ultrafast electron diffraction. Science 346, 445–448 (2014). 25342797 10.1126/science.1253779

[4] M. Först, C. Manzoni, S. Kaiser, Y. Tomioka, Y. Tokura, R. Merlin, A. Cavalleri, Nonlinear phononics as an ultrafast route to lattice control. Nat. Phys. 7, 854–856 (2011).

[5] M. Mitrano, A. Cantaluppi, D. Nicoletti, S. Kaiser, A. Perucchi, S. Lupi, P. Di Pietro, D. Pontiroli, M. Riccò, S. R. Clark, D. Jaksch, A. Cavalleri, Possible light-induced superconductivity in K_3_C_60_ at high temperature. Nature 530, 461–464 (2016). 26855424 10.1038/nature16522 PMC4820655

[6] L. Stojchevska, I. Vaskivskyi, T. Mertelj, P. Kusar, D. Svetin, S. Brazovskii, D. Mihailovic, Ultrafast switching to a stable hidden quantum state in an electronic crystal. Science 344, 177–180 (2014). 24723607 10.1126/science.1241591

[7] A. Kirilyuk, A. V. Kimel, T. Rasing, Ultrafast optical manipulation of magnetic order. Rev. Mod. Phys. 82, 2731–2784 (2010).

[8] A. De La Torre, D. M. Kennes, M. Claassen, S. Gerber, J. W. Mc Iver, M. A. Sentef, Colloquium: Nonthermal pathways to ultrafast control in quantum materials. Rev. Mod. Phys. 93, 041002 (2021).

[9] E. Beaurepaire, J.-C. Merle, A. Daunois, J.-Y. Bigot, Ultrafast spin dynamics in ferromagnetic nickel. Phys. Rev. Lett. 76, 4250–4253 (1996). 10061239 10.1103/PhysRevLett.76.4250

[10] B. Koopmans, G. Malinowski, F. Dalla Longa, D. Steiauf, M. Fähnle, T. Roth, M. Cinchetti, M. Aeschlimann, Explaining the paradoxical diversity of ultrafast laser-induced demagnetization. Nat. Mater. 9, 259–265 (2010). 20010830 10.1038/nmat2593

[11] W. You, P. Tengdin, C. Chen, X. Shi, D. Zusin, Y. Zhang, C. Gentry, A. Blonsky, M. Keller, P. M. Oppeneer, H. Kapteyn, Z. Tao, M. Murnane, Revealing the nature of the ultrafast magnetic phase transition in ni by correlating extreme ultraviolet magneto-optic and photoemission spectroscopies. Phys. Rev. Lett. 121, 077204 (2018). 30169091 10.1103/PhysRevLett.121.077204

[12] R. Mankowsky, A. Subedi, M. Först, S. O. Mariager, M. Chollet, H. T. Lemke, J. S. Robinson, J. M. Glownia, M. P. Minitti, A. Frano, M. Fechner, N. A. Spaldin, T. Loew, B. Keimer, A. Georges, A. Cavalleri, Nonlinear lattice dynamics as a basis for enhanced superconductivity in YBa_2_Cu_3_O_6.5_. Nature 516, 71–73 (2014). 25471882 10.1038/nature13875

[13] M. Trigo, M. Fuchs, J. Chen, M. P. Jiang, M. Cammarata, S. Fahy, D. M. Fritz, K. Gaffney, S. Ghimire, A. Higginbotham, S. L. Johnson, M. E. Kozina, J. Larsson, H. Lemke, A. M. Lindenberg, G. Ndabashimiye, F. Quirin, K. Sokolowski-Tinten, C. Uher, G. Wang, J. S. Wark, D. Zhu, D. A. Reis, Fourier-transform inelastic X-ray scattering from time- and momentum-dependent phonon–phonon correlations. Nat. Phys. 9, 790–794 (2013).

[14] A. H. Reid, X. Shen, P. Maldonado, T. Chase, E. Jal, P. Granitzka, K. Carva, R. K. Li, J. Li, L. Wu, T. Vecchione, T. Liu, Z. Chen, D. J. Higley, N. Hartmann, R. Coffee, J. Wu, G. L. Dakowski, W. Schlotter, H. Ohldag, Y. K. Takahashi, V. Mehta, O. Hellwig, A. Fry, Y. Zhu, J. Cao, E. E. Fullerton, J. Stöhr, P. M. Oppeneer, X. J. Wang, H. A. Dürr, Beyond a phenomenological description of magnetostriction. Nat. Commun. 9, 388 (2018). 29374151 10.1038/s41467-017-02730-7 PMC5786062

[15] S. I. Anisimov, B. L. Kapeliovich, T. L. Perel'man, Electron emission from metal surfaces exposed to ultrashort laser pulses. J. Exp. Theor. Phys. 39, 776–781 (1974) [*Zh. Eksp. Teor. Fiz.* **66**, 375–377 (1974)].

[16] P. B. Allen, Theory of thermal relaxation of electrons in metals. Phys. Rev. Lett. 59, 1460–1463 (1987). 10035240 10.1103/PhysRevLett.59.1460

[17] K. Carva, M. Battiato, P. M. Oppeneer, Ab initio investigation of the elliott-yafet electron-phonon mechanism in laser-induced ultrafast demagnetization. Phys. Rev. Lett. 107, 207201 (2011). 22181762 10.1103/PhysRevLett.107.207201

[18] M. Pickel, A. B. Schmidt, F. Giesen, J. Braun, J. Minár, H. Ebert, M. Donath, M. Weinelt, Spin-orbit hybridization points in the face-centered-cubic cobalt band structure. Phys. Rev. Lett. 101, 066402 (2008). 10.1103/PhysRevLett.101.066402. 18764479

[19] C. Dornes, Y. Acremann, M. Savoini, M. Kubli, M. J. Neugebauer, E. Abreu, L. Huber, G. Lantz, C. A. F. Vaz, H. Lemke, E. M. Bothschafter, M. Porer, V. Esposito, L. Rettig, M. Buzzi, A. Alberca, Y. W. Windsor, P. Beaud, U. Staub, D. Zhu, S. Song, J. M. Glownia, S. L. Johnson, The ultrafast Einstein–de Haas effect. Nature 565, 209–212 (2019). 30602792 10.1038/s41586-018-0822-7

[20] S. R. Tauchert, M. Volkov, D. Ehberger, D. Kazenwade, M. Evers, H. Lange, A. Donges, A. Book, W. Kreuzpaintner, U. Nowak, P. Baum, Polarized phonons carry angular momentum in ultrafast demagnetization. Nature 602, 73–77 (2022). 35110761 10.1038/s41586-021-04306-4

[21] P. Tengdin, W. You, C. Chen, X. Shi, D. Zusin, Y. Zhang, C. Gentry, A. Blonsky, M. Keller, P. M. Oppeneer, H. C. Kapteyn, Z. Tao, M. M. Murnane, Critical behavior within 20 fs drives the out-of-equilibrium laser-induced magnetic phase transition in nickel. Sci. Adv. 4, eaap9744 (2018). 29511738 10.1126/sciadv.aap9744 PMC5834307

[22] L. Waldecker, R. Bertoni, R. Ernstorfer, J. Vorberger, Electron-phonon coupling and energy flow in a simple metal beyond the two-temperature approximation. Phys. Rev. X 6, 021003 (2016).

[23] P. Maldonado, K. Carva, M. Flammer, P. M. Oppeneer, Theory of out-of-equilibrium ultrafast relaxation dynamics in metals. Phys. Rev. B 96, 174439 (2017).

[24] P. Maldonado, T. Chase, A. H. Reid, X. Shen, R. K. Li, K. Carva, T. Payer, M. Horn von Hoegen, K. Sokolowski-Tinten, X. J. Wang, P. M. Oppeneer, H. A. Dürr, Tracking the ultrafast nonequilibrium energy flow between electronic and lattice degrees of freedom in crystalline nickel. Phys. Rev. B 101, 100302(R) (2020).

[25] U. Ritzmann, P. M. Oppeneer, P. M. Maldonado, Theory of out-of-equilibrium electron and phonon dynamics in metals after femtosecond laser excitation. Phys. Rev. B 102, 214305 (2020).

[26] W. Eberhardt, E. W. Plummer, Angle-resolved photoemission determination of the band structure and multielectron excitations in Ni. Phys. Rev. B 21, 3245–3255 (1980).

[27] T. Greber, T. J. Kreutz, J. Osterwalder, Photoemission above the fermi level: The top of the MinoritydBand in nickel. Phys. Rev. Lett. 79, 4465–4468 (1997).

[28] H. S. Rhie, H. A. Dürr, W. Eberhardt, Femtosecond electron and spin dynamics in Ni/W(110) films. Phys. Rev. Lett. 90, 247201 (2003). 12857221 10.1103/PhysRevLett.90.247201

[29] J. Sánchez-Barriga, R. Ovsyannikov, J. Fink, Strong spin dependence of correlation effects in Ni due to stoner excitations. Phys. Rev. Lett. 121, 267201 (2018). 30636126 10.1103/PhysRevLett.121.267201

[30] A. Proctor, P. M. A. Sherwood, Data analysis techniques in X-ray photoelectron spectroscopy. Anal. Chem. 54, 13–19 (1982).

[31] E. Carpene, E. Mancini, C. Dallera, M. Brenna, E. Puppin, S. De Silvestri, Dynamics of electron-magnon interaction and ultrafast demagnetization in thin iron films. Phys. Rev. B 78, 174422 (2008). 10.1103/PhysRevB.78.174422.

[32] B. Y. Mueller, A. Baral, S. Vollmar, M. Cinchetti, M. Aeschlimann, H. C. Schneider, B. Rethfeld, Feedback effect during ultrafast demagnetization dynamics in ferromagnets. Phys. Rev. Lett. 111, 167204 (2013). 24182297 10.1103/PhysRevLett.111.167204

[33] C. Stamm, T. Kachel, N. Pontius, R. Mitzner, T. Quast, K. Holldack, S. Khan, C. Lupulescu, E. F. Aziz, H. A. Dürr, W. Eberhardt, Femtosecond modification of electron localization and transfer of angular momentum in nickel. Nat. Mater. 6, 740–743 (2007). 17721541 10.1038/nmat1985

[34] T. Kachel, N. Pontius, C. Stamm, M. Wietstruk, E. F. Aziz, H. A. Dürr, W. Eberhardt, F. M. F. de Groot, Transient electronic and magnetic structures of nickel heated by ultrafast laser pulses. Phys. Rev. B 80, 092404 (2009). 10.1103/PhysRevB.80.092404.

[35] K. Carva, D. Legut, P. M. Oppeneer, Influence of laser-excited electron distributions on the X-ray magnetic circular dichroism spectra: Implications for femtosecond demagnetization in Ni. Europhys. Lett. 86, 57002–57006 (2009).

[36] T. Lojewski, M. F. Elhanoty, L. Le Guyader, O. Grånäs, N. Agarwal, C. Boeglin, R. Carley, A. Castoldi, C. David, C. Deiter, F. Döring, R. Y. Engel, F. Erdinger, H. Fangohr, C. Fiorini, P. Fischer, N. Gerasimova, R. Gort, F. de Groot, K. Hansen, S. Hauf, D. Hickin, M. Izquierdo, B. E. Van Kuiken, Y. Kvashnin, C.-H. Lambert, D. Lomidze, S. Maffessanti, L. Mercadier, G. Mercurio, P. S. Miedema, K. Ollefs, M. Pace, M. Porro, J. Rezvani, B. Rösner, N. Rothenbach, A. Samartsev, A. Scherz, J. Schlappa, C. Stamm, M. Teichmann, P. Thunström, M. Turcato, A. Yaroslavtsev, J. Zhu, M. Beye, H. Wende, U. Bovensiepen, O. Eriksson, A. Eschenlohr, The interplay of local electron correlations and ultrafast spin dynamics in fcc Ni. Mater. Res. Lett. 11, 655–661 (2023).

[37] M. Dendzik, R. Patrick Xian, E. Perfetto, D. Sangalli, D. Kutnyakhov, S. Dong, S. Beaulieu, T. Pincelli, F. Pressacco, D. Curcio, S. Ymir Agustsson, M. Heber, J. Hauer, W. Wurth, G. Brenner, Y. Acremann, P. Hofmann, M. Wolf, A. Marini, G. Stefanucci, L. Rettig, R. Ernstorfer, Observation of an excitonic mott transition through ultrafast core-cum-conduction photoemission Spectroscopy. Phys. Rev. Lett. 125, 096401 (2020). 32915590 10.1103/PhysRevLett.125.096401

[38] M. Battiato, K. Carva, P. M. Oppeneer, Theory of laser-induced ultrafast superdiffusive spin transport in layered heterostructures. Phys. Rev. B 86, 024404 (2012).

[39] Y. H. Wang, D. Hsieh, E. J. Sie, H. Steinberg, D. R. Gardner, Y. S. Lee, P. Jarillo-Herrero, N. Gedik, Measurement of intrinsic dirac fermion cooling on the surface of the topological insulator Bi2Se3 using time-resolved and angle-resolved photoemission spectroscopy. Phys. Rev. Lett. 109, 127401 (2012). 23005985 10.1103/PhysRevLett.109.127401

[40] L. X. Yang, G. Rohde, T. Rohwer, A. Stange, K. Hanff, C. Sohrt, L. Rettig, R. Cortés, F. Chen, D. L. Feng, T. Wolf, B. Kamble, I. Eremin, T. Popmintchev, M. M. Murnane, H. C. Kapteyn, L. Kipp, J. Fink, M. Bauer, U. Bovensiepen, K. Rossnagel, Ultrafast modulation of the chemical potential inBaFe2As2by coherent phonons. Phys. Rev. Lett. 112, 207001 (2014).

[41] D. Kutnyakhov, R. P. Xian, M. Dendzik, M. Heber, F. Pressacco, S. Y. Agustsson, L. Wenthaus, H. Meyer, S. Gieschen, G. Mercurio, A. Benz, K. Bühlman, S. Däster, R. Gort, D. Curcio, K. Volckaert, M. Bianchi, C. Sanders, J. A. Miwa, S. Ulstrup, A. Oelsner, C. Tusche, Y. J. Chen, D. Vasilyev, K. Medjanik, G. Brenner, S. Dziarzhytski, H. Redlin, B. Manschwetus, S. Dong, J. Hauer, L. Rettig, F. Diekmann, K. Rossnagel, J. Demsar, H. J. Elmers, P. Hofmann, R. Ernstorfer, G. Schönhense, Y. Acremann, W. Wurth, Time- and momentum-resolved photoemission studies using time-of-flight momentum microscopy at a free-electron laser. Rev. Sci. Instrum. 91, 013109 (2020). 32012554 10.1063/1.5118777

[42] L. Miaja-Avila, C. Lei, M. Aeschlimann, J. L. Gland, M. M. Murnane, H. C. Kapteyn, G. Saathoff, Laser-assisted photoelectric effect from surfaces. Phys. Rev. Lett. 97, 113604 (2006). 17025885 10.1103/PhysRevLett.97.113604

[43] C. G. Ryan, E. Clayton, W. L. Griffin, S. H. Sie, D. R. Cousens, SNIP, a statistics-sensitive background treatment for the quantitative analysis of PIXE spectra in geoscience applications. Nucl. Instrum. Methods Phys. Res. B 34, 396–402 (1988).

[44] M. Morhac, J. Kliman, V. Matousek, M. Veselsky, I. Turzo, Background elimination methods for multidimensional coincidence γ-ray spectra. Nucl. Instrum. Methods Phys. Res. A Accel. Spectrom. Detect. Assoc. Equip. 401, 113–132 (1997).

[45] J. C. Fuggle, N. Mårtensson, Core-level binding energies in metals. J. Electron Spectrosc. Relat. Phenom. 21, 275–281 (1980).

[46] S. Eich, M. Plötzing, M. Rollinger, S. Emmerich, R. Adam, C. Chen, H. C. Kapteyn, M. M. Murnane, L. Plucinski, D. Steil, B. Stadtmüller, M. Cinchetti, M. Aeschlimann, C. M. Schneider, S. Mathias, Band structure evolution during the ultrafast ferromagnetic-paramagnetic phase transition in cobalt. Sci. Adv. 3, e1602094 (2017). 28378016 10.1126/sciadv.1602094 PMC5365247

[47] R. Gort, K. Bühlmann, S. Däster, G. Salvatella, N. Hartmann, Y. Zemp, S. Holenstein, C. Stieger, A. Fognini, T. U. Michlmayr, T. Bähler, A. Vaterlaus, Y. Acremann, Early stages of ultrafast spin dynamics in a 3D ferromagnet. Phys. Rev. Lett. 121, 87206 (2018). 10.1103/PhysRevLett.121.087206 30192573

