# Supplementary material for: Real-time observation of non-equilibrium phonon-electron energy and angular momentum flow in laser-heated nickel
Source: Sci Adv. 2024 Jan 31;10(5):eadj2407. doi: 10.1126/sciadv.adj2407 (PMC10830112; doi:10.1126/sciadv.adj2407)
Supplement: Supplementary file 1 — Supplementary Text Figs. S1 to S14 References [file sciadv.adj2407_sm.pdf]

Science Advances
AAAS

Supplementary Materials for

Real-time observation of non-equilibrium phonon-electron energy and angular momentum flow in laser-heated nickel

Vishal Shokeen *et al.*

Corresponding author: Hermann A. Dürr, hermann.durr@physics.uu.se

Sci. Adv. **10**, eadj2407 (2024)
DOI: 10.1126/sciadv.adj2407

This PDF file includes:

Supplementary Text
Figs. S1 to S14
References

Supplementary text

Time-dependent shift of the valence-band maximum (VBM) in tr-ARPES

Figure S1 shows the momentum dependence of the maximum energy of transient occupied valence states, E_{VBM}, defined as the energy where the photoemission intensity near E_F is reduced by half, for selected delay times after optical excitation. In the ground-state (*i.e.,* when the probe pulses arrive at the sample at a delay time, t, before the pump, *i.e.* $t < 0$), we find that the highest values of E_{VBM} occur for momenta where real bands are near E_F. This occurs when real bands cross the Fermi level, *i.e.*, for *sp*-bands in the Γ-K direction near k = 1.0 Å^{-1} (Figs. S1D, H) or for $3d$-bands in the $\Gamma - d_{11\bar{2}}$ direction near k = 1.1 Å^{-1} and 1.6 Å^{-1} in Figs. S1B, F. High values of E_{VBM} also occur near the W-point (Figs. S1A, E) when the majority $3d$-band approaches E_F without crossing it.

300 fs after laser excitation, E_{VBM} has increased at the W-point by almost 80 meV. For larger delay times E_{VBM} slowly decreases by 30 meV at 1.8 ps. A monotonic increase of E_{VBM} is observed at the *sp*-band Fermi crossing and for $3d$-states near the $K_{01\bar{1}}$-point. Such a significant momentum dependence of electronic excitations has not been reported before for Ni *(11, 21, 28)* or other ferromagnets *(46, 47).* The significantly different shift of E_{VBM} observed for different k-points excludes space charge effects as a cause of this behavior. For other materials, an increase of E_{VBM} has been attributed to a change of the global chemical potential, *i.e.,* to increased band occupations *(39, 40).*

Core-level measurements

To corroborate the observed chemical potential shifts in the valence band, we also measured time-resolved $3p$ core level photoemission spectra for the same fluence conditions. The unpumped spectrum in Fig. S7A is averaged between time delays -0.4 ps < t < 0.2 ps. It clearly shows spin-orbit-split peaks whose binding energy was determined by fitting two Voigt functions for each core-level. The background for the core-level spectra has been calculated using the statistics-sensitive non-linear iterative peak-clipping (SNIP) algorithm *(43, 44).* We obtain values for binding energies of the spin-orbit-split $3p_{1/2}$ and $3p_{3/2}$ peaks of 68.0 and 66.2 eV with a width of 1.7 and 1.8 eV, respectively which agree well with the reports in the literature *(45).*

Upon pumping at a pump fluence of 3.7 mJ/cm^2 we observe a slight attenuation of the amplitude as shown in Fig. S12. This amplitude reduction could be due to enhanced scattering of photoemitted electrons with the increase of phonon population. The change in peak binding energy with pump-probe time delay is illustrated in Fig. S7B for a time-delay of 1.8 ps. In order to reduce the number of fit parameters we keep the ratio of peak amplitudes; peak width and spin-orbit splitting the same at all time delays.

Figure S7B also shows the comparison of the measured difference spectra with two fit conditions which are 2% (1.7%) reduction in amplitude and 12 meV (15 meV) binding-energy shifts towards the Fermi level. The change in the amplitude and peak shift have different signatures in the difference photoemission spectra with absorptive and dispersive behavior respectively. As shown in Fig. S7B, 15 meV shift and 1.7% amplitude decrease results in better fit (gray plot) in the slope around 66 eV but introduces a positive feature around 65 eV whereas blue fits better at 65 eV at the cost of poor fit in the slope.

Correction of the Light Assisted Photoemission Effect (LAPE)

The LAPE is a transient process occurring only during the temporal overlap of intense infra-red (IR) pump and XUV probe *(42).* LAPE appears as a step in the photoemission intensity near Fermi level and is described as the laser (IR) dressing of the emitted photoelectrons in the continuum modifying the photoemission spectrum as shown in Fig S9. Above the Fermi level additional photoemission intensity appears due to the absorption of an IR photon by an XUV emitted photoelectron. The above-Fermi intensity distribution roughly follows the direct photoemission spectrum at lower binding energy shifted by the laser photon energy of 1.2 eV. The change in intensity above Fermi level (red line in Fig. S9A) relative to the ground-state photoemission spectra (dashed black lines) is a sensitive measure of the temporal duration of pump and probe pulses (see Fig. S10). We obtain values of 227±2 fs for the convoluted pump and probe pulse duration. For analyzing the spectral lineshape of photoemission spectra in temporal pump-probe overlap it is necessary to subtract the LAPE induced lineshape changes. Since we only analyze the lineshape in the vicinity of the Fermi level it is possible to remove the LAPE modifications as indicated in Fig. S9B. The LAPE corrected spectrum shown in red in Fig. 9B is very similar to the unpumped spectrum justifying the correction procedure in the binding energy range of ±1 eV around the Fermi level where significant effects due to pump laser are expected to occur. This correction procedure is then repeated for other time delays where LAPE is present (i.e. for delay times between -0.2 ps < t < 0.2 ps) to extract the pump induced dynamical signal.

Fitting valence photoemission spectra

We used the fit functions described in Eqs. (S1-S3) for modelling the photoemission signal for k-dependent density of states. The fit function generally includes the product of density of states with Fermi-Dirac distribution convoluted with a gaussian function in Eq. (S1). The Fermi-Dirac distribution determines the filling of the density of states while the gaussian convolution mimics the instrumental resolution. The evolution of the Fermi function for different k-points provides information about the change in the chemical potential and electronic temperature induced broadening around the Fermi level. The measured photoemission intensity is then

$$I(E') = \left(f_{dos}(E') * f_{FD}(E', \mu, T_e)\right) \otimes G(E', \delta) \qquad \text{(S1)}$$

where $E' = E - E_F$, is the electron energy relative to the Fermi level, E_F. f_{dos}, f_D, μ and T_e represent the density of states, Fermi-Dirac distribution, chemical potential and electronic temperature respectively. $\otimes$ represents the convolution of a gaussian function, *G*, with δ as its FWHM describing the energy resolution of the experiment.

To extract the dynamics of exchange splitting from *k*-points with minority and majority *d*-bands, the density of states for up and down spin *d*-bands is produced by using Lorentzian functions Eq. (S2) as

$$f_{d-dos} = l_{min}(E', x_0 + E_{ex}, dx) + l_{maj}(E', x_0, dx) \qquad \text{(S2)}$$

with x_0, E_{ex} *and* dx representing the energy position, exchange splitting and width of Lorentzian functions for minority and majority *d*-bands. The electronic temperature for these k-points are the values extracted from the fitting of *sp*-bands. The delay-

independent background for *d*-bands is extracted using the Shirley method (*30*). The extracted energy shift of majority band for different *k*-points has been evaluated using two exponential functions convolved with temporal resolution (δ_t) shown in Eq. (S3) as

$$\Delta E_{maj} = a * \left(\exp\left(-\frac{t}{\tau_{demag}}\right) - \exp\left(-\frac{t}{\tau_{remag}}\right)\right) \otimes \text{erf}\,(\delta_t) \qquad \text{(S3)}$$

where τ_{demag} and τ_{remag} are demagnetization and remagnetization time constants, respectively, erf is the error function.

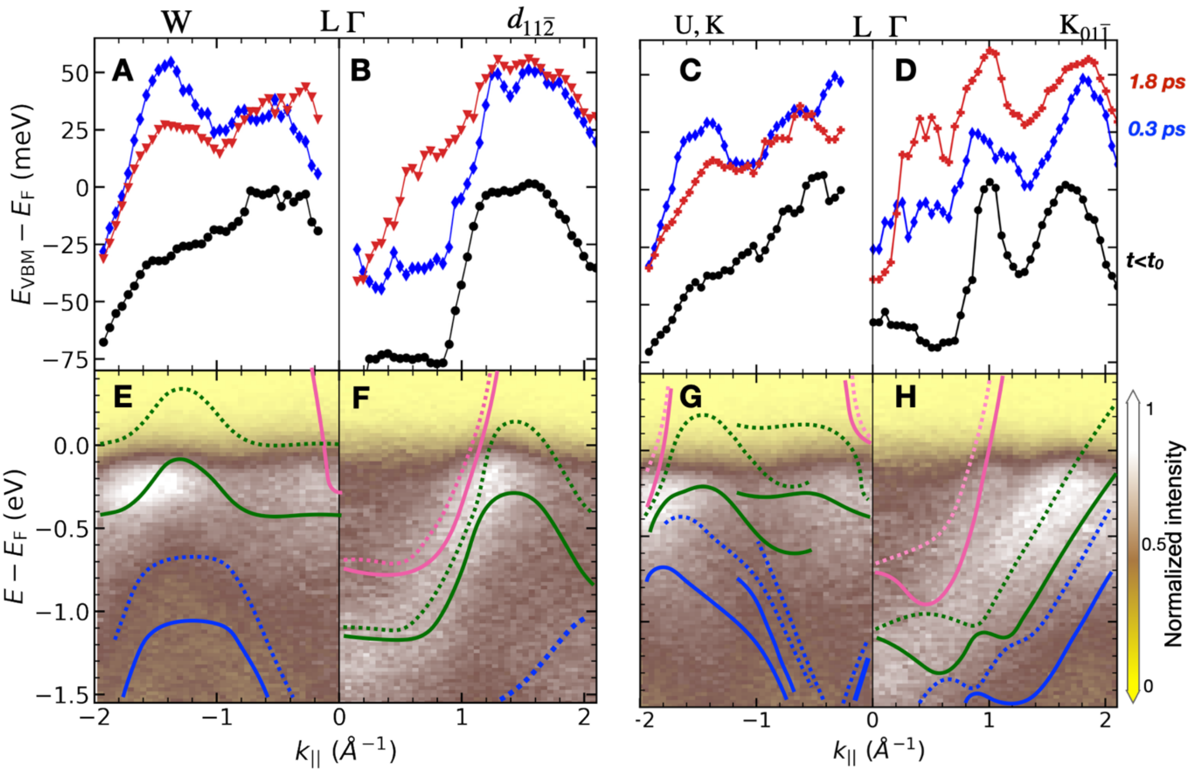

Fig. S1. Changes in the photoemission signal from states near the Fermi level, E_F. (**A-D**) Energy position of the top-most occupied valence-band electronic state, E_{VBM}, relative to the Fermi level, E_F, along the respective directions for various pump-probe time delays. E_{VBM} was chosen as the inflection point of data such as the ones shown in Fig. 2. (**E-H**) Momentum resolved ARPES intensity (color shading) and calculated electronic state dispersions (lines) along L-W, Γ-$d_{11\bar{2}}$, L-U and Γ-K Brillouin-zone directions (see Fig. 1C), respectively. Data were obtained for a pump fluence of 3.7 mJ/cm^2.

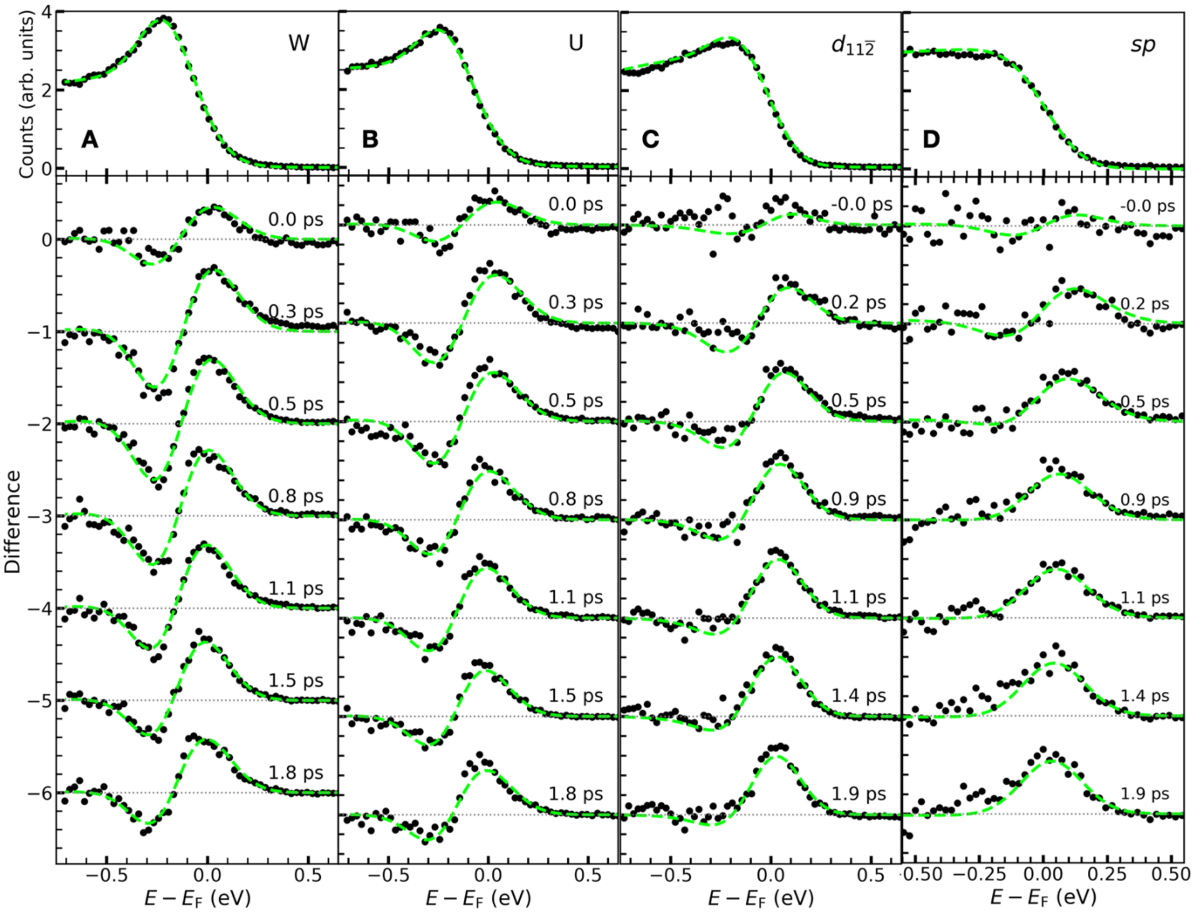

Fig. S2. Tr-ARPES from *k*-points at 3.7 mJ/cm^2 pump fluence. Shown are the ARPES data at (**A**) W, (**B**) U, (**C**) $d_{11\bar{2}}$ and (**D**) *sp* points obtained before laser pump (top panels) as well as difference spectra at the indicated pump-probe time delays integrated over ±150 fs (waterfall plots in bottom panels) for the indicated k-points averaged over the k-regions shown in Fig. S1. Data were obtained for a pump fluence of 3.7 mJ/cm^2. Regions in (k_x, k_y) over which the data of this figure were averaged are indicated in Fig. S5.

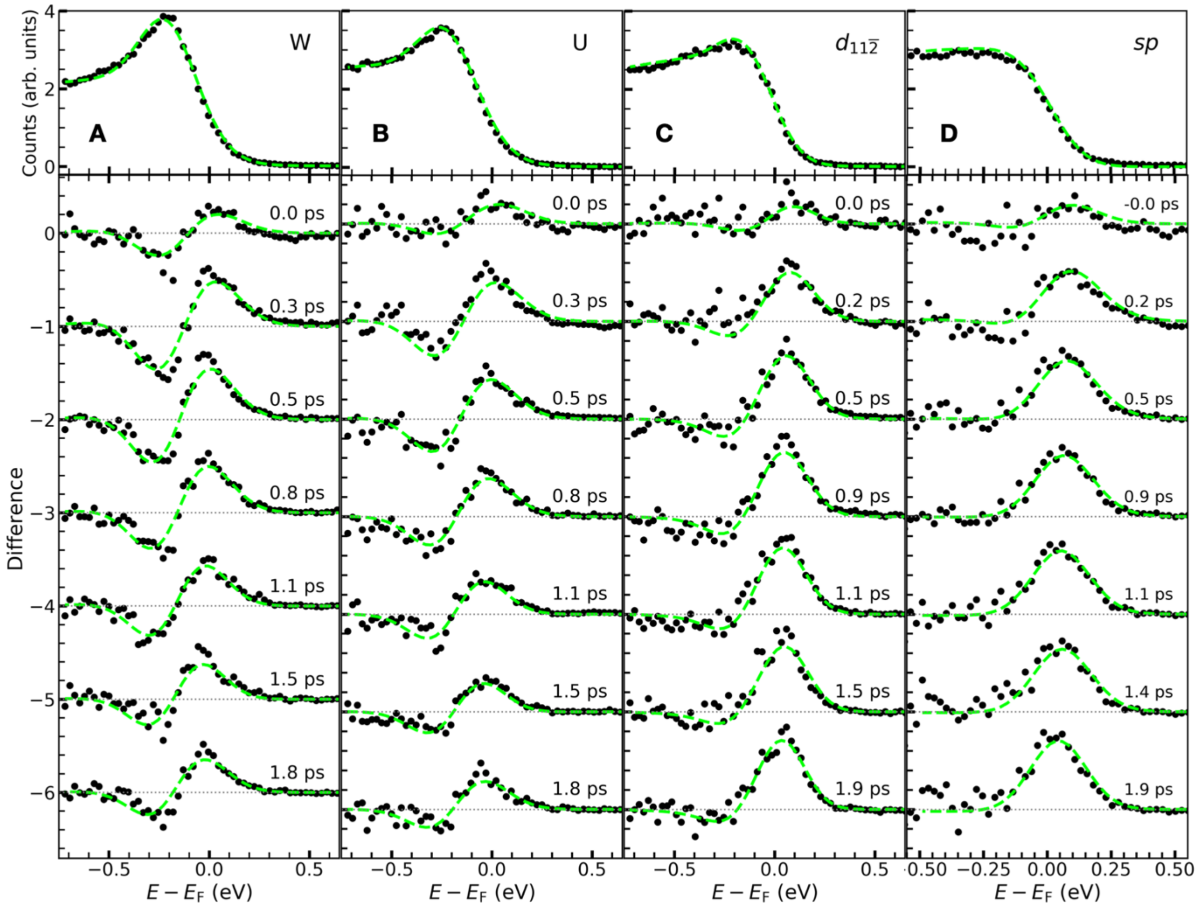

Fig. S3. Tr-ARPES from *k*-points at 2.2 mJ/cm² pump fluence. Shown are the ARPES data obtained at (**A**) W, (**B**) U, (**C**) $d_{11\bar{2}}$ and (**D**) *sp* points before laser pump (top panels) as well as difference spectra at the indicated pump-probe time delays integrated over ±150 fs (waterfall plots in bottom panels) for the indicated k-points averaged over the k-regions shown in Fig. S1. Data were obtained for a pump fluence of 2.2 mJ/cm². Regions in (k_x, k_y) over which the data of this figure were averaged are indicated in Fig. S5.

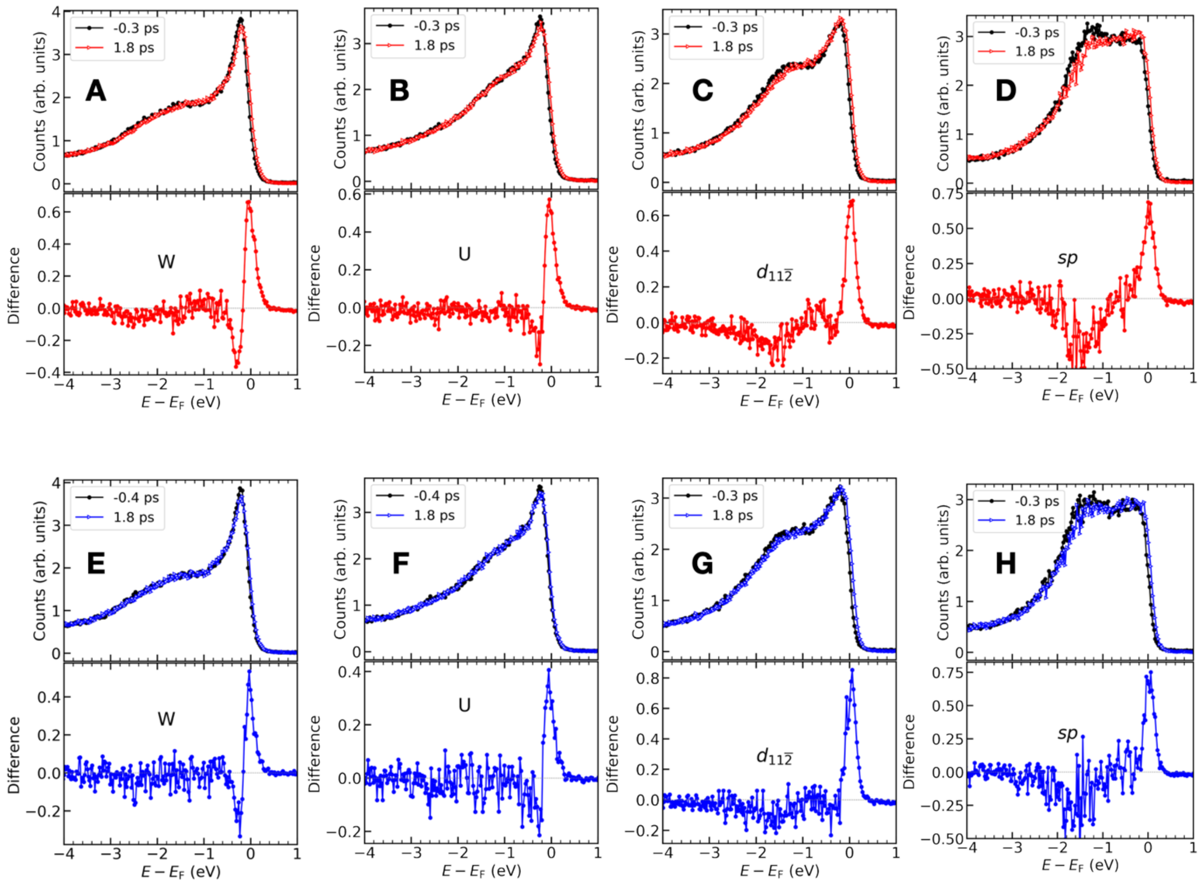

Fig. S4. Tr-ARPES from W, U, $d_{11\bar{2}}$ and sp-points for a broader BE range from 1 to -4 eV around E_F. (**A-D**) Pump fluence 3.7 mJ/cm^2, (**E-H**) 2.2 mJ/cm^2. The black lines and symbols represent the energy spectra for t < -0.3 ps and colored (red, blue) lines and symbols are for a delay of 1.8 ps. All data were averaged over ±300 fs. Regions in (k_x, k_y) over which the data of this figure were averaged are indicated in Fig. S5.

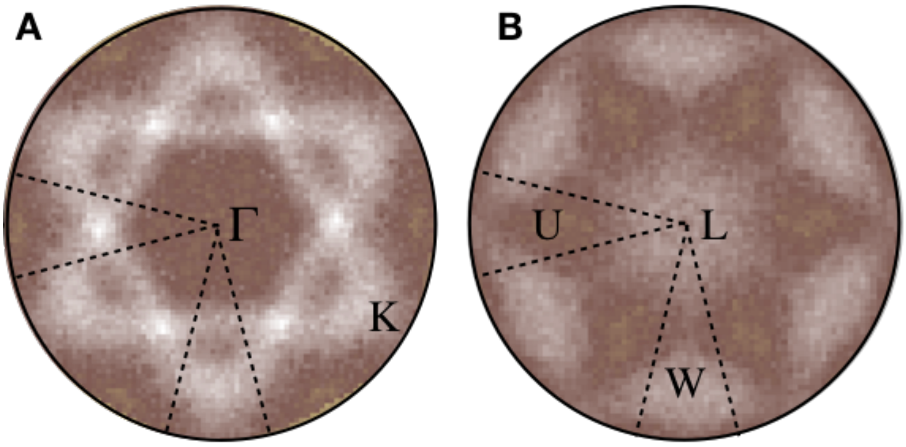

Fig. S5. ARPES from states near the Fermi level in Ni(111). (**A**) photoemission intensity from states near E_F in the Γ-K plane of the Ni(111) Brillouin zone (see Fig. 1C) (**B**) same as in (**A**) but for the L-U-W plane in Fig. 1C. The shown regions bounded by dotted lines along different *k*-directions are used for the analysis in Fig. 2 and Fig S2.

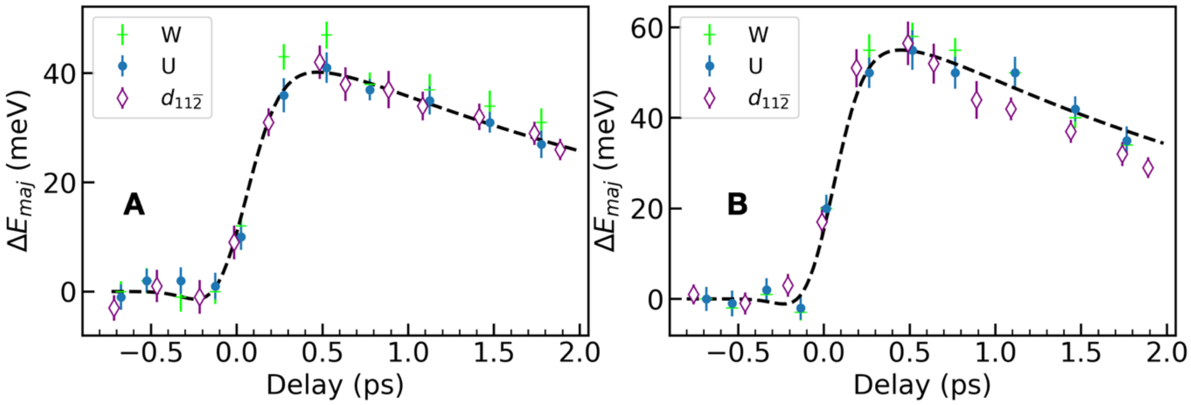

Fig. S6. Temporal evolution of the 3*d* exchange splitting at different *k*-points. Energy positions, ΔE_{maj}, of the majority spin-bands for tr-ARPES spectra at the W, U and $d_{11\bar{2}}$ -points obtained by fits as shown in Fig. 4 of the main paper for pump fluences of (**A**) 2.2 mJ/cm^2 and (**B**) 3.7 mJ/cm^2. Dashed black lines represent the fits to the data according to Eq. (S3) with demagnetization times, τ_{demag}, of 205±50 fs and 200±70 fs, and remagnetization times, τ_{remag}, of 3.0±0.5 and 2.9±0.7 ps for 2.2 and 3.7 mJ/cm^2, respectively.

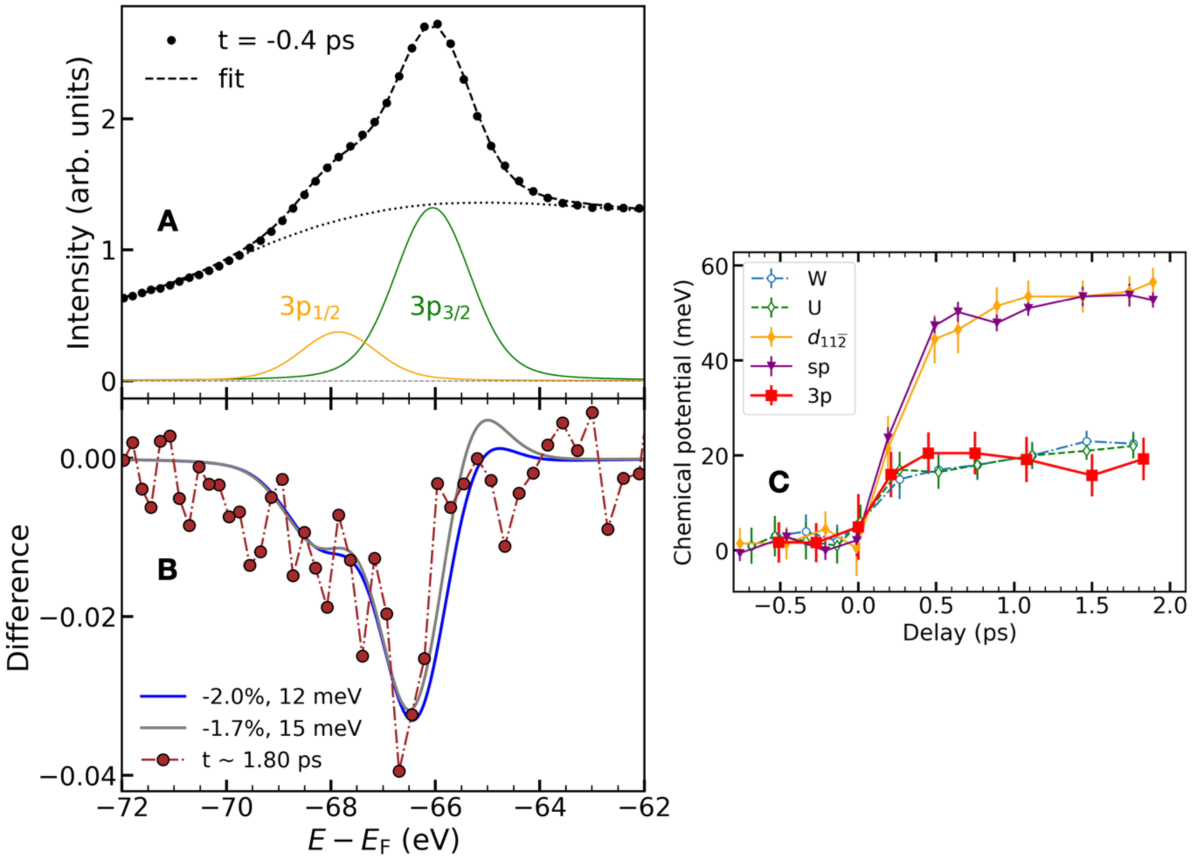

Fig. S7. Temporal evolution of Ni(111) 3*p* core-level photoemission. (**A**) 3*p* core-level photoemission spectra measured with 204 eV shown in black markers. The dotted black line is the calculated background using the SNIP method described in the supplementary text. The dashed line is the fit obtained using two Voigt functions representing the spin-orbit-split $3p_{1/2}$ and $3p_{3/2}$ core levels (colored solid lines). (**B**) Difference between pumped and unpumped core-level photoemission spectra at a pump fluence of 3.7 mJ/cm^2 (dash-dotted line and circles). Blue and gray lines are the fits obtained for two conditions as described in the supplementary text. (**C**) Temporal evolution of the core-level-shift (red line and squares) towards the Fermi level compared with chemical potential change for the indicated *k*-points.

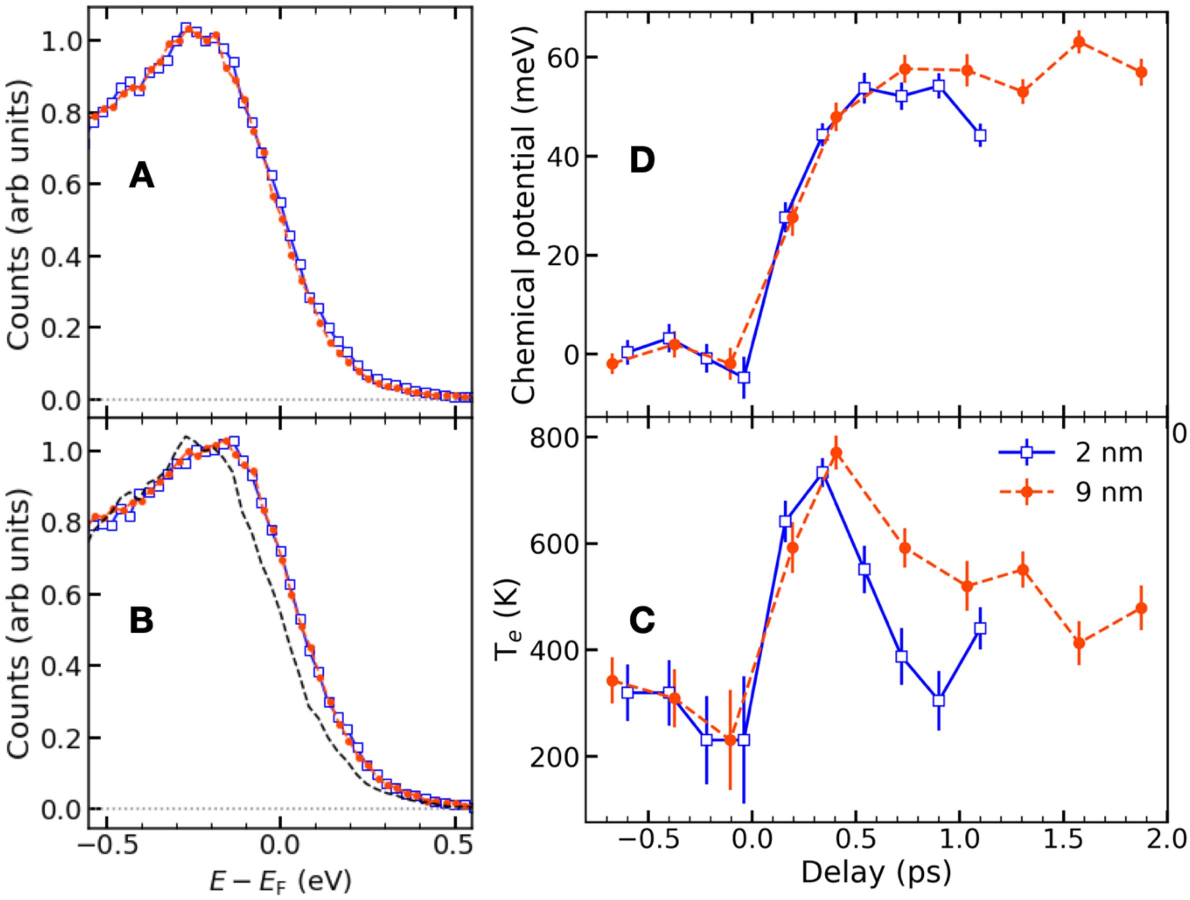

Fig. S8. Tr-ARPES of Ni(111)/W(110) of different Ni thicknesses. (**A**) Unpumped angle-integrated photoemission spectra taken for Ni films of 2 nm thickness (blue lines and open squares) and 9 nm thickness at 205 eV photon energy (red lines and solid circles). At 205 eV photon energy similar parts of the Ni BZ are probed as at 74 eV. (**B**) Pumped angle-integrated photoemission spectra taken for Ni films of 2 and 9 nm thickness as in (**A**) averaged over ±0.3 ps around a pump-probe time delay of 1 ps. (**C**) Measured electron temperatures, T_e, vs. delay time for the 2 and 9 nm thick Ni films. The pump fluence of the 2 nm film was 3.7 mJ/cm^2 as in the main paper. The pump fluence of the 9 nm film was adjusted to achieve a similar maximum T_e value, i.e. a similar deposited pump laser energy. (**D**) Chemical potential change for the two Ni thicknesses with pump-probe delay.

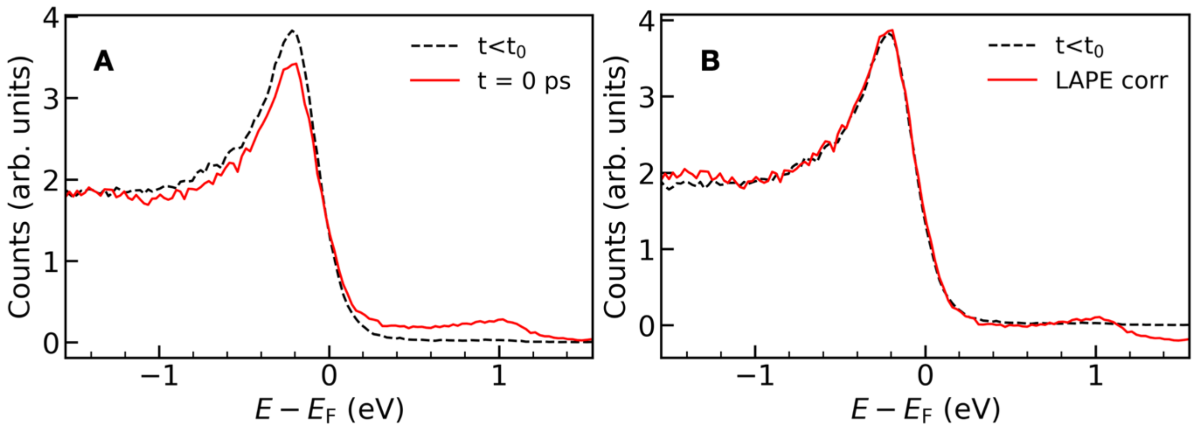

Fig. S9. Correction of the Light Assisted Photoemission Effect (LAPE). A) The black dashed line is the unpumped photoemission spectrum and the red solid line is the pumped photoemission spectrum with pump and probe pulses in temporal overlap. The deviation of both spectra is due to the LAPE effect. (**B**) The pumped spectrum after LAPE correction (solid red line) is compared with black unpumped spectrum (dashed black line). LAPE correction is done by first subtracting an offset to remove the step above the Fermi level and then multiplying the remaining spectrum by a factor to match it to the unpumped spectrum.

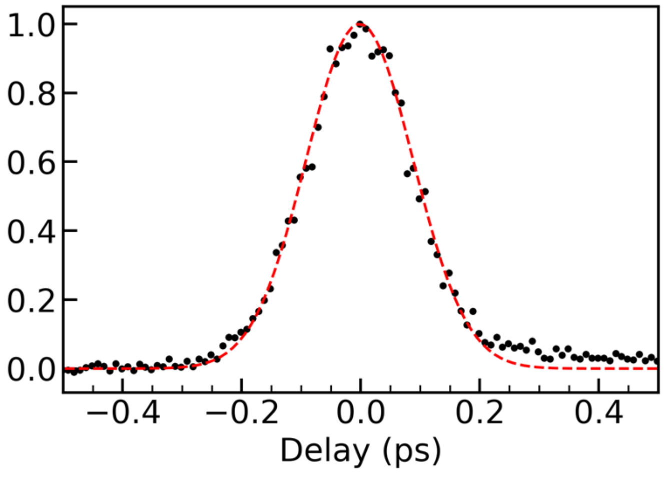

Fig. S10. Temporal instrumental resolution. The black markers represent the photoemission signal above the Fermi level in the LAPE region (see Fig. S9 A). The red dashed line is a gaussian fit with a FWHM of 227±2 fs.

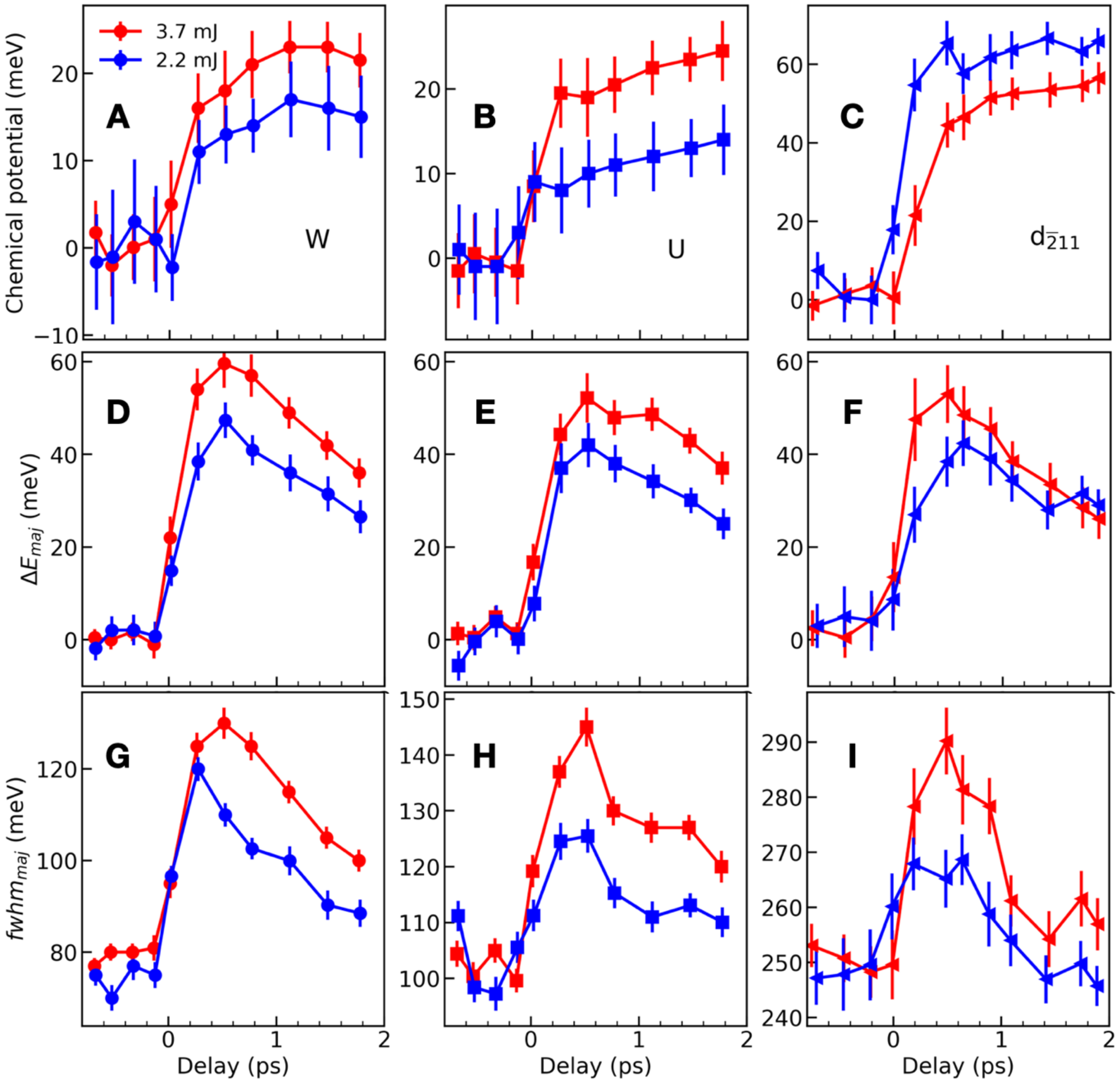

Fig. S11. Extracted fit parameters at W, U and $d_{11\bar{2}}$ points with exchange split d-bands for 3.7 (red) and 2.2 (blue) mJ/cm^2 pump fluence. **(A-C)** chemical potential shift, **(D-F)** shift of majority band due to exchange collapse. **(G-I)** full width half maximum (FWHM) broadening of majority bands due to laser excitation as a function of pump probe delay.

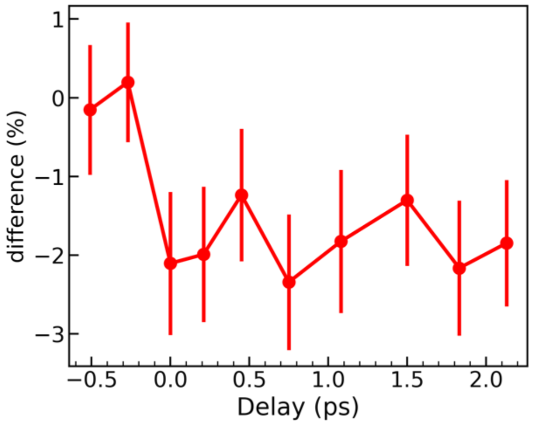

Fig. S12. Laser induced reduction of 3*p* peaks. This figure shows the relative change in the mean amplitude of the 3p peaks for different pump-probe delays. There is a reduction in the amplitude of 3p peaks by ~1.7-2% after laser excitation.

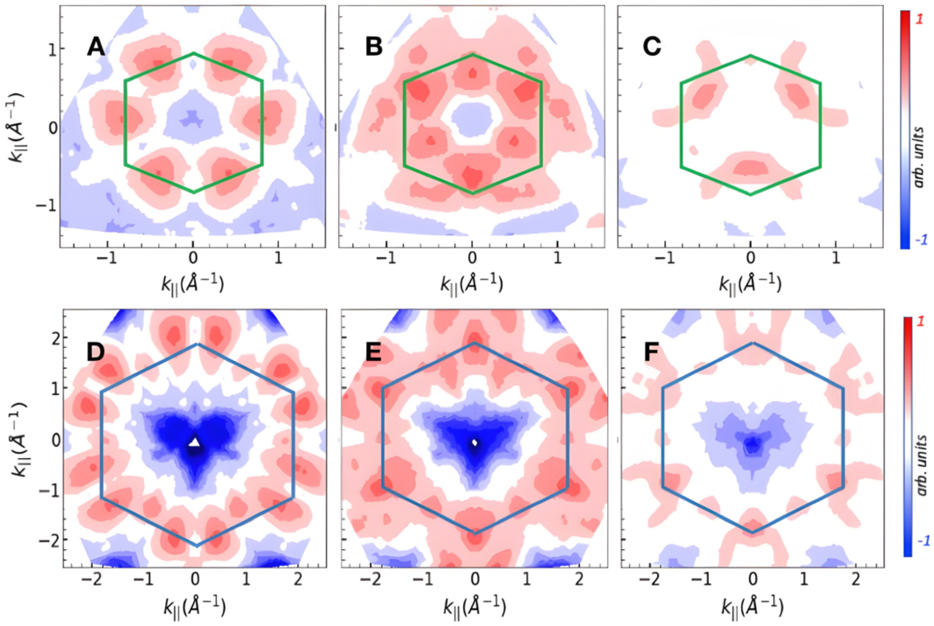

Fig. S13. Calculated transient phonon occupations. This figure shows the differences of transient phonon occupation relative to those in thermal equilibrium 1 ps after fs laser excitation in the L-W-U (**A-C**) and Γ-K (**D-F**) planes of the Ni Brillouin zone (see Fig. 1B). Panels (**A**, **B**, **D**, **E**) show phonons with transverse polarization while (**C**, **F**) display longitudinal phonon polarization.

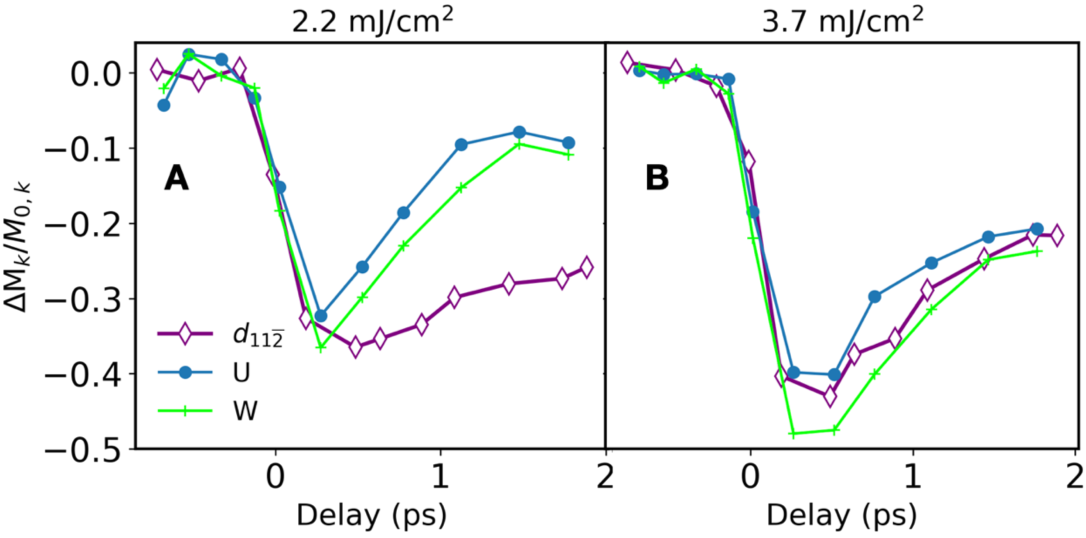

Fig. S14. Momentum-dependent magnetic moment evolution. This figure shows the changes of the magnetic moments at the respective *k*-points vs. pump-probe time delay for pump fluences of (**A**) 2.2 mJ/cm^2 and (**B**) 3.7 mJ/cm^2. $\frac{\Delta M_k}{M_k}$ is defined as $\frac{\Delta M_k}{M_k} = \frac{N_{maj}-N_{min}}{N_{0,maj}-N_{0,min}}$, where $N_{maj,min}$ are the occupations on majority and minority *d*-bands determined from Fig. 3 and N_0 represents the respective occupation numbers before laser excitation.

REFERENCES

1. A. Kimel, A. Kirilyuk, P. A. Usachev, R. V. Pisarev, A. M. Balbashov, T. Rasing, Ultrafast non-thermal control of magnetization by instantaneous photomagnetic pulses. *Nature* **435**, 655–657 (2005).

2. F. Schmitt, P. S. Kirchmann, U. Bovensiepen, R. G. Moore, L. Rettig, M. Krenz, J.-H. Chu, N. Ru, L. Perfetti, D. H. Lu, M. Wolf, I. R. Fisher, Z.-X. Shen, Transient electronic structure and melting of a charge density wave in $TbTe_3$. *Science* **321**, 1649–1652 (2008).

3. V. R. Morrison, R. P. Chatelain, K. L. Tiwari, A. Hendaoui, A. Bruhacs, M. Chaker, B. J. Siwick, A photoinduced metal-like phase of monoclinic VO2 revealed by ultrafast electron diffraction. *Science* **346**, 445–448 (2014).

4. M. Först, C. Manzoni, S. Kaiser, Y. Tomioka, Y. Tokura, R. Merlin, A. Cavalleri, Nonlinear phononics as an ultrafast route to lattice control. *Nat. Phys.* **7**, 854–856 (2011).

5. M. Mitrano, A. Cantaluppi, D. Nicoletti, S. Kaiser, A. Perucchi, S. Lupi, P. Di Pietro, D. Pontiroli, M. Riccò, S. R. Clark, D. Jaksch, A. Cavalleri, Possible light-induced superconductivity in K_3C_{60} at high temperature. *Nature* **530**, 461–464 (2016).

6. L. Stojchevska, I. Vaskivskyi, T. Mertelj, P. Kusar, D. Svetin, S. Brazovskii, D. Mihailovic, Ultrafast switching to a stable hidden quantum state in an electronic crystal. *Science* **344**, 177–180 (2014).

7. A. Kirilyuk, A. V. Kimel, T. Rasing, Ultrafast optical manipulation of magnetic order. *Rev. Mod. Phys.* **82**, 2731–2784 (2010).

8. A. De La Torre, D. M. Kennes, M. Claassen, S. Gerber, James W. Mc Iver, M. A. Sentef, Colloquium: Nonthermal pathways to ultrafast control in quantum materials. *Rev. Mod. Phys.* **93**, 041002 (2021).

9. E. Beaurepaire, J.-C. Merle, A. Daunois, J.-Y. Bigot, Ultrafast spin dynamics in ferromagnetic nickel. *Phys. Rev. Lett.* **76**, 4250–4253 (1996).

10. B. Koopmans, G. Malinowski, F. Dalla Longa, D. Steiauf, M. Fähnle, T. Roth, M. Cinchetti, M. Aeschlimann, Explaining the paradoxical diversity of ultrafast laser-induced demagnetization. *Nat. Mater.* **9**, 259–265 (2010).

11. W. You, P. Tengdin, C. Chen, X. Shi, D. Zusin, Y. Zhang, C. Gentry, A. Blonsky, M. Keller, P. M. Oppeneer, H. Kapteyn, Z. Tao, M. Murnane, Revealing the nature of the ultrafast magnetic phase transition in ni by correlating extreme ultraviolet magneto-optic and photoemission spectroscopies. *Phys. Rev. Lett.* **121**, 077204 (2018).

12. R. Mankowsky, A. Subedi, M. Först, S. O. Mariager, M. Chollet, H. T. Lemke, J. S. Robinson, J. M. Glownia, M. P. Minitti, A. Frano, M. Fechner, N. A. Spaldin, T. Loew, B. Keimer, A. Georges, A. Cavalleri, Nonlinear lattice dynamics as a basis for enhanced superconductivity in $YBa_2Cu_3O_{6.5}$. *Nature* **516**, 71–73 (2014).

13. M. Trigo, M. Fuchs, J. Chen, M. P. Jiang, M. Cammarata, S. Fahy, D. M. Fritz, K. Gaffney, S. Ghimire, A. Higginbotham, S. L. Johnson, M. E. Kozina, J. Larsson, H. Lemke, A. M. Lindenberg, G. Ndabashimiye, F. Quirin, K. Sokolowski-Tinten, C. Uher, G. Wang, J. S. Wark, D. Zhu, D. A. Reis, Fourier-transform inelastic X-ray scattering from time- and momentum-dependent phonon–phonon correlations. *Nat. Phys.* **9**, 790–794 (2013).

14. A. H. Reid, X. Shen, P. Maldonado, T. Chase, E. Jal, P. Granitzka, K. Carva, R. K. Li, J. Li, L. Wu, T. Vecchione, T. Liu, Z. Chen, D. J. Higley, N. Hartmann, R. Coffee, J. Wu, G. L. Dakowski, W. Schlotter, H. Ohldag, Y. K. Takahashi, V. Mehta, O. Hellwig, A. Fry, Y. Zhu, J. Cao, E. E. Fullerton, J. Stöhr, P. M. Oppeneer, X. J. Wang, H. A. Dürr, Beyond a phenomenological description of magnetostriction. *Nat. Commun.* **9**, 388 (2018).

15. S. I. Anisimov, B. L. Kapeliovich, T. L. Perel'man, Electron emission from metal surfaces exposed to ultrashort laser pulses. *J. Exp. Theor. Phys.* **39**, 776–781 (1974) [*Zh. Eksp. Teor. Fiz.* **66**, 375–377 (1974)].

16. P. B. Allen, Theory of thermal relaxation of electrons in metals. *Phys. Rev. Lett.* **59**, 1460–1463 (1987).

17. K. Carva, M. Battiato, P. M. Oppeneer, Ab initio investigation of the elliott-yafet electron-phonon mechanism in laser-induced ultrafast demagnetization. *Phys. Rev. Lett.* **107**, 207201 (2011).

18. M. Pickel, A. B. Schmidt, F. Giesen, J. Braun, J. Minár, H. Ebert, M. Donath, M. Weinelt, Spin-orbit hybridization points in the face-centered-cubic cobalt band structure. *Phys. Rev. Lett.* **101**, 066402 (2008). https://doi.org/10.1103/PhysRevLett.101.066402.

19. C. Dornes, Y. Acremann, M. Savoini, M. Kubli, M. J. Neugebauer, E. Abreu, L. Huber, G. Lantz, C. A. F. Vaz, H. Lemke, E. M. Bothschafter, M. Porer, V. Esposito, L. Rettig, M. Buzzi, A. Alberca, Y. W. Windsor, P. Beaud, U. Staub, D. Zhu, S. Song, J. M. Glownia, S. L. Johnson, The ultrafast Einstein–de Haas effect. *Nature* **565**, 209–212 (2019).

20. S. R. Tauchert, M. Volkov, D. Ehberger, D. Kazenwade, M. Evers, H. Lange, A. Donges, A. Book, W. Kreuzpaintner, U. Nowak, P. Baum, Polarized phonons carry angular momentum in ultrafast demagnetization. *Nature* **602**, 73–77 (2022).

21. P. Tengdin, W. You, C. Chen, X. Shi, D. Zusin, Y. Zhang, C. Gentry, A. Blonsky, M. Keller, P. M. Oppeneer, H. C. Kapteyn, Z. Tao, M. M. Murnane, Critical behavior within 20 fs drives the out-of-equilibrium laser-induced magnetic phase transition in nickel. *Sci. Adv.* **4**, eaap9744 (2018).

22. L. Waldecker, R. Bertoni, R. Ernstorfer, J. Vorberger, Electron-phonon coupling and energy flow in a simple metal beyond the two-temperature approximation. *Phys. Rev. X* **6**, 021003 (2016).

23. P. Maldonado, K. Carva, M. Flammer, P. M. Oppeneer, Theory of out-of-equilibrium ultrafast relaxation dynamics in metals. *Phys. Rev. B* **96**, 174439 (2017).

24. P. Maldonado, T. Chase, A. H. Reid, X. Shen, R. K. Li, K. Carva, T. Payer, M. Horn von Hoegen, K. Sokolowski-Tinten, X. J. Wang, P. M. Oppeneer, H. A. Dürr, Tracking the ultrafast nonequilibrium energy flow between electronic and lattice degrees of freedom in crystalline nickel. *Phys. Rev. B* **101**, 100302(R) (2020).

25. U. Ritzmann, P. M. Oppeneer, P. M. Maldonado, Theory of out-of-equilibrium electron and phonon dynamics in metals after femtosecond laser excitation. *Phys. Rev. B* **102**, 214305 (2020).

26. W. Eberhardt, E. W. Plummer, Angle-resolved photoemission determination of the band structure and multielectron excitations in Ni. *Phys. Rev. B* **21**, 3245–3255 (1980).

27. T. Greber, T. J. Kreutz, J. Osterwalder, Photoemission above the fermi level: The top of the MinoritydBand in nickel. *Phys. Rev. Lett.* **79**, 4465–4468 (1997).

28. H. S. Rhie, H. A. Dürr, W. Eberhardt, Femtosecond electron and spin dynamics in Ni/W(110) films. *Phys. Rev. Lett.* **90**, 247201 (2003).

29. J. Sánchez-Barriga, R. Ovsyannikov, J. Fink, Strong spin dependence of correlation effects in Ni due to stoner excitations. *Phys. Rev. Lett.* **121**, 267201 (2018).

30. A. Proctor, P. M. A. Sherwood, Data analysis techniques in X-ray photoelectron spectroscopy. *Anal. Chem.* **54**, 13–19 (1982).

31. E. Carpene, E. Mancini, C. Dallera, M. Brenna, E. Puppin, S. De Silvestri, Dynamics of electron-magnon interaction and ultrafast demagnetization in thin iron films. *Phys. Rev. B* **78**, 174422 (2008). https://doi.org/10.1103/PhysRevB.78.174422.

32. B. Y. Mueller, A. Baral, S. Vollmar, M. Cinchetti, M. Aeschlimann, H. C. Schneider, B. Rethfeld, Feedback effect during ultrafast demagnetization dynamics in ferromagnets. *Phys. Rev. Lett.* **111**, 167204 (2013).

33. C. Stamm, T. Kachel, N. Pontius, R. Mitzner, T. Quast, K. Holldack, S. Khan, C. Lupulescu, E. F. Aziz, H. A. Dürr, W. Eberhardt, Femtosecond modification of electron localization and transfer of angular momentum in nickel. *Nat. Mater.* **6**, 740–743 (2007).

34. T. Kachel, N. Pontius, C. Stamm, M. Wietstruk, E. F. Aziz, H. A. Dürr, W. Eberhardt, F. M. F. de Groot, Transient electronic and magnetic structures of nickel heated by ultrafast laser pulses. *Phys. Rev. B* **80**, 092404 (2009). https://doi.org/10.1103/PhysRevB.80.092404.

35. K. Carva, D. Legut, P. M. Oppeneer, Influence of laser-excited electron distributions on the X-ray magnetic circular dichroism spectra: Implications for femtosecond demagnetization in Ni. *Europhys. Lett.* **86**, 57002–57006 (2009).

36. T. Lojewski, M. F. Elhanoty, L. Le Guyader, O. Grånäs, N. Agarwal, C. Boeglin, R. Carley, A. Castoldi, C. David, C. Deiter, F. Döring, R. Y. Engel, F. Erdinger, H. Fangohr, C. Fiorini, P. Fischer, N. Gerasimova, R. Gort, F. de Groot, K. Hansen, S. Hauf, D. Hickin, M. Izquierdo, B. E. Van Kuiken, Y. Kvashnin, C.-H. Lambert, D. Lomidze, S. Maffessanti, L. Mercadier, G. Mercurio, P. S. Miedema, K. Ollefs, M. Pace, M. Porro, J. Rezvani, B. Rösner, N. Rothenbach, A. Samartsev, A. Scherz, J. Schlappa, C. Stamm, M. Teichmann, P. Thunström, M. Turcato, A. Yaroslavtsev, J. Zhu, M. Beye, H. Wende, U. Bovensiepen, O. Eriksson, A. Eschenlohr, The interplay of local electron correlations and ultrafast spin dynamics in fcc Ni. *Mater. Res. Lett.* **11**, 655–661 (2023).

37. M. Dendzik, R. Patrick Xian, E. Perfetto, D. Sangalli, D. Kutnyakhov, S. Dong, S. Beaulieu, T. Pincelli, F. Pressacco, D. Curcio, S. Ymir Agustsson, M. Heber, J. Hauer, W. Wurth, G. Brenner, Y. Acremann, P. Hofmann, M. Wolf, A. Marini, G. Stefanucci, L. Rettig, R. Ernstorfer, Observation of an excitonic mott transition through ultrafast core-cum-conduction photoemission Spectroscopy. *Phys. Rev. Lett.* **125**, 096401 (2020).

38. M. Battiato, K. Carva, P. M. Oppeneer, Theory of laser-induced ultrafast superdiffusive spin transport in layered heterostructures. *Phys. Rev. B* **86**, 024404 (2012).

39. Y. H. Wang, D. Hsieh, E. J. Sie, H. Steinberg, D. R. Gardner, Y. S. Lee, P. Jarillo-Herrero, N. Gedik, Measurement of intrinsic dirac fermion cooling on the surface of the topological insulator Bi2Se3 using time-resolved and angle-resolved photoemission spectroscopy. *Phys. Rev. Lett.* **109**, 127401 (2012).

40. L. X. Yang, G. Rohde, T. Rohwer, A. Stange, K. Hanff, C. Sohrt, L. Rettig, R. Cortés, F. Chen, D. L. Feng, T. Wolf, B. Kamble, I. Eremin, T. Popmintchev, M. M. Murnane, H. C. Kapteyn, L. Kipp, J. Fink, M. Bauer, U. Bovensiepen, K. Rossnagel, Ultrafast modulation of the chemical potential inBaFe2As2by coherent phonons. *Phys. Rev. Lett.* **112**, 207001 (2014).

41. D. Kutnyakhov, R. P. Xian, M. Dendzik, M. Heber, F. Pressacco, S. Y. Agustsson, L. Wenthaus, H. Meyer, S. Gieschen, G. Mercurio, A. Benz, K. Bühlman, S. Däster, R. Gort, D. Curcio, K. Volckaert, M. Bianchi, C. Sanders, J. A. Miwa, S. Ulstrup, A. Oelsner, C. Tusche, Y. J. Chen, D. Vasilyev, K. Medjanik, G. Brenner, S. Dziarzhytski, H. Redlin, B. Manschwetus, S. Dong, J. Hauer, L. Rettig, F. Diekmann, K. Rossnagel, J. Demsar, H. J. Elmers, P. Hofmann, R. Ernstorfer, G. Schönhense, Y.

Acremann, W. Wurth, Time- and momentum-resolved photoemission studies using time-of-flight momentum microscopy at a free-electron laser. *Rev. Sci. Instrum.* **91**, 013109 (2020).

42. L. Miaja-Avila, C. Lei, M. Aeschlimann, J. L. Gland, M. M. Murnane, H. C. Kapteyn, G. Saathoff, Laser-assisted photoelectric effect from surfaces. *Phys. Rev. Lett.* **97**, 113604 (2006).

43. C. G. Ryan, E. Clayton, W. L. Griffin, S. H. Sie, D. R. Cousens, SNIP, a statistics-sensitive background treatment for the quantitative analysis of PIXE spectra in geoscience applications. *Nucl. Instrum. Methods Phys. Res. B* **34**, 396–402 (1988).

44. M. Morhac, J. Kliman, V. Matousek, M. Veselsky, I. Turzo, Background elimination methods for multidimensional coincidence γ-ray spectra. *Nucl. Instrum. Methods Phys. Res. A Accel. Spectrom. Detect. Assoc. Equip.* **401**, 113–132 (1997).

45. J. C. Fuggle, N. Mårtensson, Core-level binding energies in metals. *J. Electron Spectrosc. Relat. Phenom.* **21**, 275–281 (1980).

46. S. Eich, M. Plötzing, M. Rollinger, S. Emmerich, R. Adam, C. Chen, H. C. Kapteyn, M. M. Murnane, L. Plucinski, D. Steil, B. Stadtmüller, M. Cinchetti, M. Aeschlimann, C. M. Schneider, S. Mathias, Band structure evolution during the ultrafast ferromagnetic-paramagnetic phase transition in cobalt. *Sci. Adv.* **3**, e1602094 (2017).

47. R. Gort, K. Bühlmann, S. Däster, G. Salvatella, N. Hartmann, Y. Zemp, S. Holenstein, C. Stieger, A. Fognini, T. U. Michlmayr, T. Bähler, A. Vaterlaus, Y. Acremann, Early stages of ultrafast spin dynamics in a 3D ferromagnet. *Phys. Rev. Lett.* **121**, 87206 (2018).
